# The Allstruder: an open syringe extruder for every 3D printer

**DOI:** 10.1016/j.ohx.2026.e00827

**Published:** 2026-08-18

**Authors:** Thomas Hinton, Riley Patten, Antonio J. PereiraTavares, Cody Crosby, Daniel J. Shiwarski

**Affiliations:** a Department of Biomedical Engineering, Tufts University, Boston, MA, United States; b Department of Biomedical Engineering, The University of Texas at Austin, Austin, TX, United States; c Department of Physics, Southwestern University, Georgetown, TX, United States; d Department of Bioengineering, University of Pittsburgh, Pittsburgh, PA, United States; e Vascular Medicine Institute, University of Pittsburgh Medical Center, Pittsburgh, PA 15213, United States; f Department of Medicine, University of Pittsburgh, Pittsburgh, PA 15213, United States

**Keywords:** Bioprint, 3D print, Additive manufacturing, Syringe pump, Extruder, Printer modification

## Abstract

The Allstruder is a modular syringe pump and extruder system engineered to combine high performance, broad compatibility, and exceptional ease of use. It is a compelling alternative to existing material extrusion tools across bioprinting, embedded 3D printing, food printing, ceramics, and multi-material applications. Validated across multiple laboratories and use cases, the Allstruder has demonstrated reliable performance and adaptability with a wide range of materials, syringe sizes, and 3D printer platforms. Designed specifically for additive manufacturing, it is configurable for nearly any extrusion-based 3D printer, supports syringes from 0.1 to 100  mL, and relies exclusively on standard, off-the-shelf components. Its architecture emphasizes adaptability through modular mounts and interchangeable syringe adapters, allowing rapid reconfiguration for different tools, materials, and workflows. With a simple, durable, and modifiable design, the Allstruder supports both novice users and experienced researchers, providing a robust platform for reproducible and high-resolution syringe-based extrusion in scientific, educational, and industrial environments.

## Graphical abstract

**Figure:**
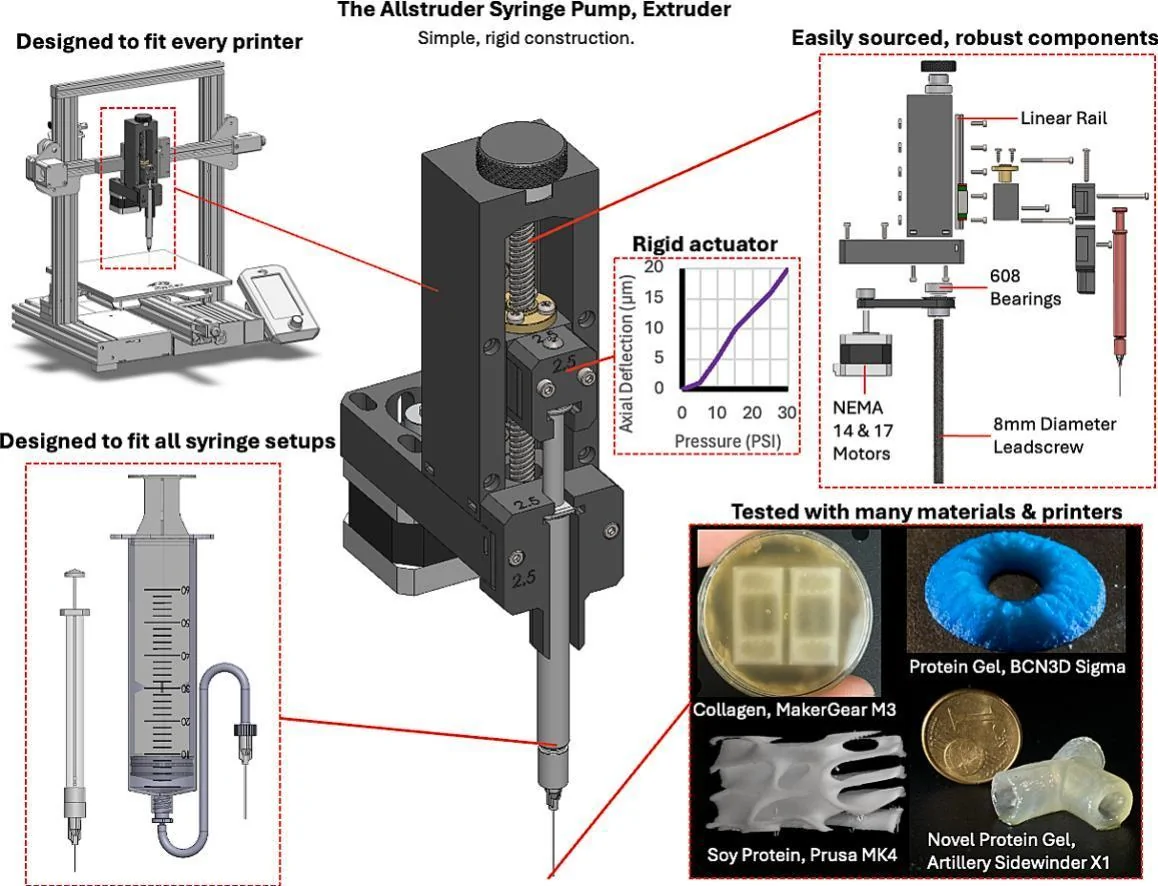
The Allstruder syringe pump extruder. Overview of the Allstruder, an open-source syringe pump extruder built for simple, rigid construction and broad compatibility across desktop 3D printers and syringe formats. The device is assembled from easily sourced, robust components, including a linear rail, NEMA 14 and 17 motors, and an 8 mm diameter leadscrew. Its rigid actuator limits axial deflection and improves extrusion performance. The Allstruder has been validated for multiple materials, direct ink writing, FRESH embedded printing, and across printer platforms such as: collagen (MakerGear M3), protein gel (BCN3D Sigma), soy protein (Prusa MK4), and a novel protein gel (Artillery Sidewinder X1).

Specifications table

**Table:** 

Hardware name	*The Allstruder Syringe Pump Extruder*
Subject area	• Engineering and materials science
Hardware type	• Other: Additive manufacturing of fluids and pastes
Closest commercial analog	*CellInk BioX*
Open source license	*CERN OHL v2*
Cost of hardware	*51 USD*
Source file repository	*https://doi.org/10.5281/zenodo.15670349*
Secondary repository	*https://github.com/**hintonthomas**/Allstruder*

## 1 Hardware in context

### 1.1 A gap in syringe-based extrusion

The increasing demand for high performance 3D printing across bioprinting, food printing, ceramics, electronics, and soft robotics has exposed the limitations of existing extrusion technologies. Commercial systems typically fall into two categories: volumetrically accurate syringe pumps that lack responsiveness, or pressure-based syringe extruders that are fast to develop pressure but imprecise[1]. Both are expensive, difficult to customize, and rarely compatible with the open, modular workflows used in many research labs and educational settings. This gap poses a technical barrier that drives many labs to reinvent hardware to meet the same unmet need, failing to account for reproducibility and accessibility between users. A shared, high-performance, and interoperable standard for a syringe extruder is how open-source innovation could unite various additive manufacturing communities.

### 1.2 A fragmented landscape of DIY solutions

Despite these limitations, no widely adopted open-source alternative has emerged. The rise of accessible desktop thermoplastic 3D printers has inspired a wave of DIY and open-source fluid extrusion systems, many of which are creative and resourceful[2], [3], [4], [5], [6], [7], [8], [9], [10], [11], [12], [13], [14], [15], [16], [17], [18], [19], [20], [21], [22], [23], [24]. However, most are tailored for specific needs, materials, or machines, and few are engineered to generalize across use cases, users, and environments. The result is a cluttered design space with dozens of extruders that partially solve a problem but rarely scale beyond a single lab or community. As a result, labs are frequently forced to tailor their workflows to the constraints of a specific extruder, locking themselves into siloed methodologies that limit reproducibility, interoperability, and shared progress. Such fragmentation can elicit reinvention of existing solutions and slow research progress; for example, a graduate student should not devote significant time to developing an in-house extruder for their desired application. To address this common pitfall of lab-locked innovation, an open-source design should excel across performance, accessibility, and adaptability.

### 1.3 Building on prior open-source innovations

While most DIY syringe extruders have fallen short of general-purpose adoption, several designs stand out as foundational and have meaningfully advanced the field:

• The Replistruder series brought affordable, lightweight, precise, and retractable syringe extrusion to embedded/FRESH 3D bioprinting and has been adapted to many other bioprinting applications, offering early compatibility between standard 3D printers and glass syringes. The Replistruder 3 set a benchmark in accessibility and performance, becoming widely adopted in academic labs. The Replistruder 4 reinforced reliability with improved mechanics and off-the-shelf parts, while The Replistruder 5 shifted toward high performance and multi-material applications. As the most widely cited and remixed open-source syringe extruder family, Replistruders shaped the field through community-led iteration and design-for-additive principles [25], [26], [27], [28], [29], [30], [31], [32], [33].
• The Large Volume Extruder (LVE) was designed to handle high-volume bioprinting using 50 mL syringes, offering continuous flow for larger prints and embedded constructs. The LVE expanded the range of printable volumes and made it easier to work with bulkier pastes such as clays, but its size made it challenging to fabricate with and attach to average desktop 3D printers [34].
• The Enderstruder optimized for cost and simplicity. By adapting the ubiquitous and affordable *Creality Ender 3* 3D printer into a capable fluid printer with minimal investment, it democratized entry-level bioprinting and inspired further community experimentation [21].
• The oldest platform, the open-source Fab@Home 1 and 2 allowed many labs to experiment with primitive direct ink write pathing and concepts like extruder retraction during printing [35].

These designs and many others helped demonstrate the value of open-source hardware in advancing syringe-based extrusion. However, no single extruder has yet achieved the necessary balance of performance, adaptability, and accessibility to unify researchers across disciplines and reduce the fragmentation of the field of fluids additive manufacturing.

### 1.4 The Allstruder: A general-purpose extrusion platform

The Allstruder was developed not just to improve performance, but to serve as an open, generalizable, robust, and user-friendly platform for syringe-based extrusion and additive manufacturing. It was designed in direct response to the proliferation of incompatible extruder projects, with the goal of supporting reproducible research, community-driven innovation, and standardization.

Key features include:

• Compatible with most extrusion-based 3D printers
• Precise actuator uses a rigid, low-friction drive system
• Versatile configurations for multi-material, high volume, high resolution, lightweight, etc.
• Material-agnostic: designed to print ceramics, foods, bioinks, etc.
• Syringe-agnostic: adapters support glass and plastic syringes from 0.1 mL to 100 mL
• Motor-agnostic: designed to work with any NEMA 14/17 motor and potentially others
• Slim form factor supports multi-material setups and tight-fit applications
• Low center of mass reduces extruder moment forces during high-speed printing
• Minimal complexity: requires basic tools and standard hardware
• Easy to make, install, configure, use, service, and modify
• 3D-printable components are optimized for fused filament fabrication (FFF/FDM)
• Tested performance across a wide range of materials and methods
• Actively being developed by a team of enthusiasts and academics

The Allstruder has already been tested across multiple labs and application areas, including printing of hydrogels, pastes, and viscous fluids prone to bubble retention or non-Newtonian flow. Its modular design allows it to be easily mounted on diverse printer architectures and reconfigured for specialized use cases.

### 1.5 Toward standardization and shared infrastructure

Beyond its mechanical design, the Allstruder represents an effort to standardize a common foundation for the next generation of syringe-based printing. By offering an extrusion system that is broadly compatible, reproducible, and open to modification, we hope to reduce redundancy in future design efforts and encourage community collaboration rather than reinvention.

Our aim is to consolidate lessons from past tools within a single platform that is ready for adoption and extension. The Allstruder is offered as a freely accessible, adaptable, high-performance solution that invites participation from students, educators, researchers, and advanced users alike, and may serve as a unifying base for reproducible manufacturing of complex fluids.

## 2 Hardware description

**Fig. 1:**
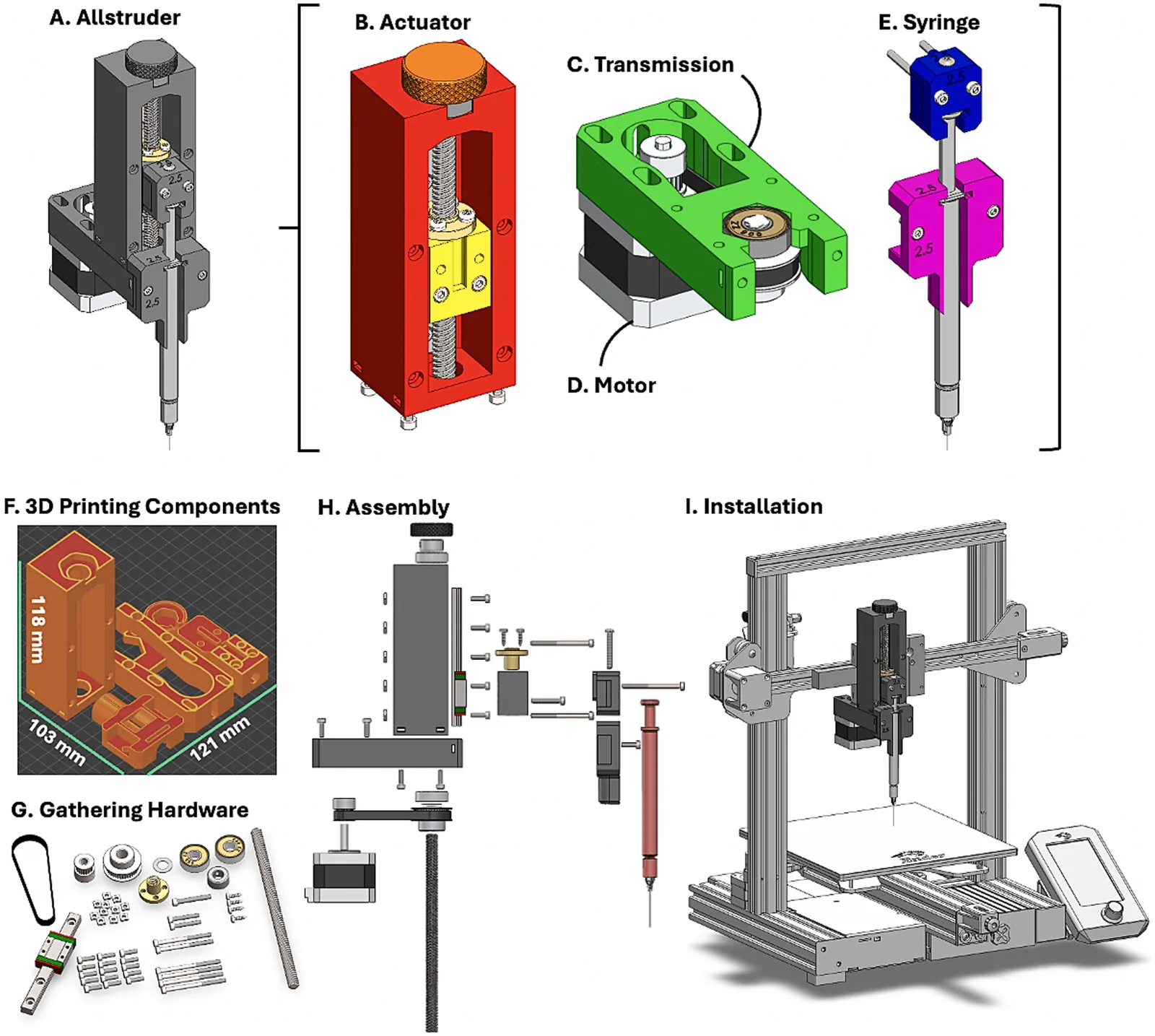
Allstruder. A) An isometric view of the Allstruder render which is broken into 4 primary components. B) The actuator is leadscrew-driven and supported by a linear rail as well as ball bearings on either end of the leadscrew. The brass leadscrew nut is affixed to the linear rail truck by a 3D printed “pusher block” (yellow). The entire actuator is housed within the 3D printed “frame” (red). A 3D printed “knob” (orange) caps the top end of the leadscrew. C) The transmission consists of a belt coupling between the shaft of the motor and the actuator’s lead screw. The transmission is housed within the 3D printed “motor mount” (green). D) The motor attaches to the bottom of the 3D printed “motor mount”. E) The syringe is attached to the Allstruder by adapters – the “pusher” adapter (blue) connects to the syringe plunger, and the “holder” adapter (purple) connects to the syringe barrel. F) 3D Printable components for the Allstruder can be 3D printed using any 3D printer with a 125 x 105 x 120 mm or larger print volume. G) Hardware for the Allstruder consists of standard, mass-produced components such as M3 bolts. H) Assembly of the Allstruder can be accomplished mostly within a single plane with the Allstruder resting flat on its side. I) When complete, the Allstruder can operate standalone as a syringe pump or installed as an extruder on a 3D printer. (For interpretation of the references to colour in this figure legend, the reader is referred to the web version of this article.)

### 2.1 Actuator

The actuator (Fig. 1B) forms the backbone of the Allstruder and provides the structural framework for mounting to a 3D printer. It consists of a 3D printed frame that houses a leadscrew-driven pusher block, which is guided along a precision linear rail. The leadscrew is supported by ball bearings on both ends, ensuring smooth, stable motion. The pusher block is supported by the linear rail truck and features a keyslot and through-holes for attaching the syringe pusher adapter. This design enables controlled depression/retraction of the syringe plunger. The actuator comes in two size variants: a regular version with a 66 mm stroke and an extended version with a 116 mm stroke, accommodating different syringe volumes and motion requirements.

### 2.2 Transmission

The transmission (Fig. 1C) is housed within a 3D printed motor mount and consists of a timing belt connecting the motor output to the actuator’s lead screw. The motor mounts to the motor mount using slotted holes, which allow it to slide backward for tensioning the timing belt. This indirect drive setup, when used with a quality, fiber-backed timing belt such as 2GT, combines straightforward tensioning and backlash reduction with smooth torque transfer. The motor mount also includes two front-facing bosses with square nut slots for mounting the syringe holder adapter. The transmission is currently available in two versions optimized for different NEMA motor sizes: NEMA 17 (requires a 180 mm belt) and NEMA 14 (requires a 120 mm belt) and is easily configurable to other motor mounting patterns and sizes.

### 2.3 Motor

The motor (Fig. 1D) serves as the driving force behind the Allstruder. Currently, NEMA 17 and NEMA 14 stepper motors are supported, but any motor that fits within the space underneath the motor mount can be made to work.

### 2.4 Syringe

The syringe interface (Fig. 1E) allows the Allstruder to handle a wide range of plunger-driven syringe sizes, including reusable gastight Hamilton syringes from 0.1 to 25 mL and disposable plastic syringes from 3 to 100 mL. Each syringe is connected using a pair of 3D printed adapters: the “pusher” attaches to the syringe plunger and mounts to the pusher block, while the “holder” attaches to the syringe barrel and secures to the motor mount. This modular adapter system enables easy swapping of syringes and maintains alignment during extrusion. Each adapter set is designed to position the syringe as close as possible to the actuator assembly, which serves as the primary mechanical reference. This mechanical optimization minimizes deflection in the pusher, motor mount bosses, and adapter interfaces, thereby reducing positional inaccuracies during extrusion.

### 2.5 Key aspects of the hardware

• The Allstruder offers an upgrade for most DIY syringe pumping, extruding, and 3D printing. It can replace an aging Replistruder or LVE and offer greatly expanded functionality.
• As seen in the Bill of Materials, the cost per Allstruder hovers around 50 USD ($), depending on part sources. When building it the first time, the initial cost is higher because few components are available in exact needed quantity. Choosing premium components will produce a more expensive device, and per unit cost can be significantly reduced when buying components in bulk to assemble multiple Allstruders.
• The Allstruder is compatible with any liquid extrusion-based printing method, material, syringe style, or motor. A syringe warmer has been developed for materials like chocolate, and a syringe cooler is in development.
• Assembly (Fig. 6, Fig. 7, Fig. 8, Fig. 9, Fig. 10, Fig. 11) requires standard tools and hardware such as metric Allen wrenches and bolts
• Tested on 12 + popular 3D printers/bioprinters (many shown in Fig. 12A)
• Possible configurations support a variety of uses without needing modification
  - Large ink volumes
  - High pressure extrusion
  - High resolution extrusion
  - High speed extrusion
  - Coaxial extrusion
  - Multiple extruder setups (multiple materials)
  - Bowden extruder setups (syringe connected to nozzle via tubing)

### 2.6 Comparing Allstruder to other DIY syringe pump extruders

**Fig. 2:**
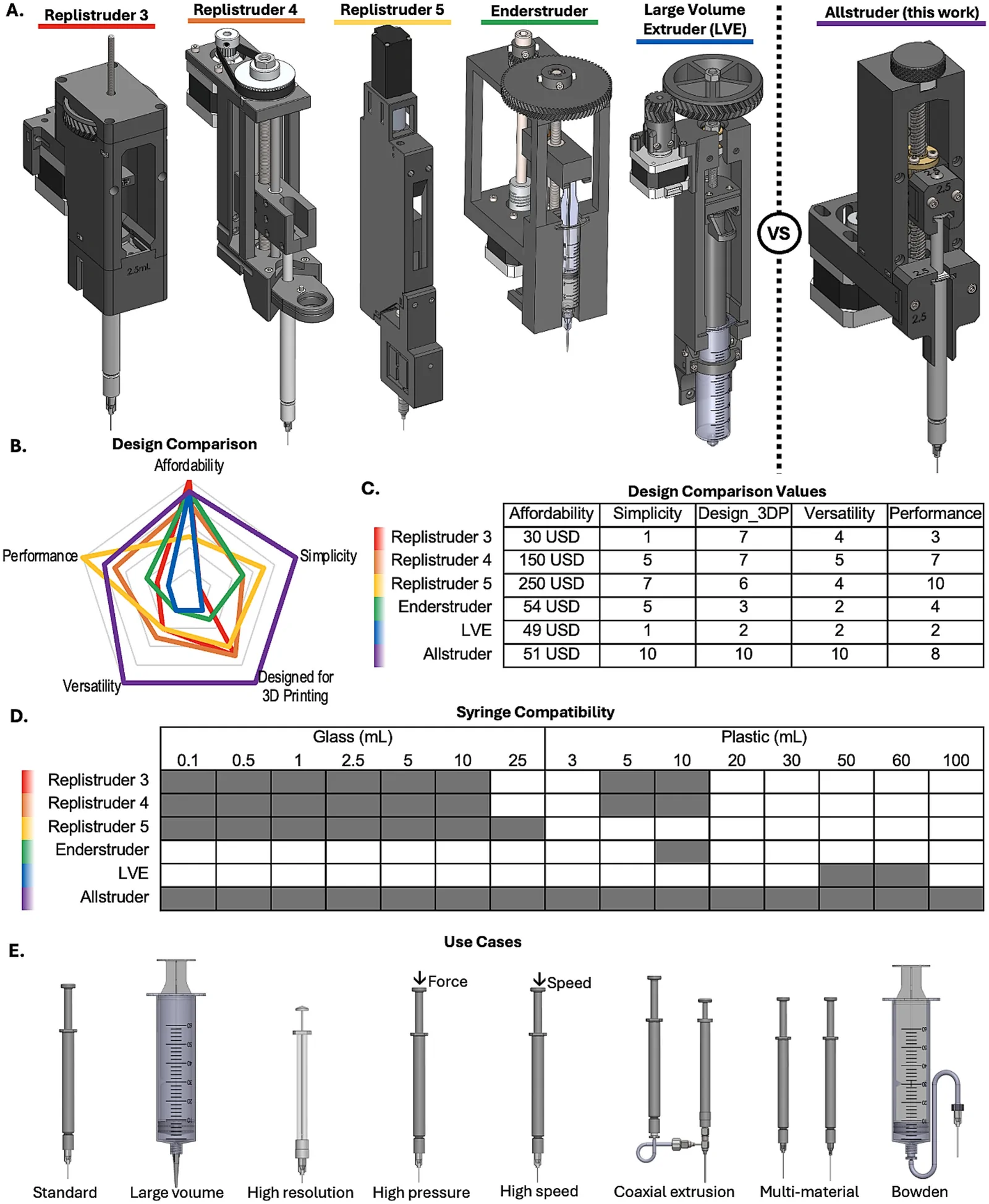
Comparison to Existing Open Source Extruders. A) The Replistruders, Enderstruder, and Large Volume Extruder inspired the design of the Allstruder. B) A radar plot showing how prior DIY extruders and the Allstruder differ with respect to affordability, performance, simplicity, versatility, and design for 3D printing. C) The table of scores attributed to the DIY extruders along each of the axes of comparison. D) The Allstruder supports all syringes used by other DIY syringe pump extruders. E) The Allstruder supports all syringe pump extruder use-cases.

In developing the Allstruder, we combined our experience designing the published open-source extruders shown in Fig. 2A with insights gained from working with commercial bioprinting systems from brands such as Allevi (3D Systems), CellInk (BICO), RegenHU, and Puredyne® (Viscotec). We also gathered insight from several unpublished or proprietary extruder systems (e.g., Fig. 4B), some of which are specialized for manufacturing, high-pressure, high-volume, or high-speed applications. The goal was to integrate the most effective features from across the ecosystem into a design that maximized utility without introducing unnecessary complexity or restricting users.

To better understand the limitations of existing DIY syringe extruders and identify unmet needs, we developed a five-metric framework to evaluate their utility and guide Allstruder design (Fig. 2B). We believe affordability, performance, simplicity, versatility, and design for 3D printing capture important design principles for open usability and reproducibility in open-source syringe pump extruders.

• Affordability refers to the total cost of producing and using the device, including printed parts and mechanical components.
• Performance describes the mechanical quality of the actuator, focusing on rigidity, smooth motion, and minimal backlash. This includes how well the mechanism resists friction and misalignment, both of which affect accuracy and precision during extrusion.
• Simplicity considers how intuitive and reliable the device is to build, configure, and operate. This includes the ease of changing syringes, avoiding assembly mistakes, and whether the documentation helps users succeed without trial and error. The best designs are straightforward to assemble with a low rate of error. Many of them include step-by-step video guides, clear written instructions, and answers to common questions.
• Versatility measures how broadly the extruder can be used, whether it accepts different syringe sizes, works with multiple printer types, supports various motors, and adapts to a wide range of applications without needing redesign.
• Designed for 3D printing refers to how well the parts are optimized for FFF. This includes printability on standard machines, support-free geometries, optimizations countering part deformations, and overall ease of production.

Together, these five metrics offer a structured way to compare syringe pump designs and assess their usefulness in real-world printing contexts. To apply this framework consistently, we decomposed each of the five metrics into specific, measurable sub-criteria and scored every extruder against them. The complete rubric, sub-criteria, and per-device scores are provided in the accompanying spreadsheet [Supplementary File / Zenodo deposit], so that each score can be traced to its basis and the same rubric applied to extruders not considered here. The resulting scores are summarized in Fig. 2B-C and are best understood not as rankings but as a map of deliberate design trade-offs.

These scores also make clear that each prior extruder represents a deliberate optimization for a particular corner of the design space rather than a failure to reach an ideal, and the differences in Fig. 2B-C largely reflects those choices and the era in which each tool was made. The Replistruder 3 was produced when no comparable open syringe extruder existed, and it prioritized affordability, printability, and performance on low-cost hardware. In expert hands it produced some of the best embedded bioprinting of its time, but it required considerable expertise and was not intended to be simple to interpret, which is why it scores well on affordability and performance but low on simplicity. The Replistruder 4 responded to those limitations with a structural change, introducing off-the-shelf metallic elements into an otherwise 3D-printed mechanism to trade fully printed construction for the stiffness of aluminum and steel. This hybrid approach preserved much of the accessibility and prototypability of the series while delivering a substantial gain in performance, and it was supported by thorough, widely circulated documentation. The Replistruder 5 carried that trajectory to its conclusion as a lightweight, compact, high-precision extruder in which nearly all of the cost is invested in a premium leadscrew, rail, and motor. It ranks highest in mechanical performance because it is optimized entirely around glass syringes and a narrow, high-quality configuration for cartridge-style, multi-toolhead systems, and this same specialization is why it scores lower on versatility and affordability, by intent rather than by oversight. The Enderstruder occupies the opposite corner, forgoing optimization and breadth to maximize simplicity and accessibility for a single popular low-cost printer, while the Large Volume Extruder accepts the resolution ceiling imposed by a 50 mL syringe, which offsets the benefit of better hardware, in exchange for the large volumes and low-cost materials such as clays and alginates that made it broadly useful.

Taken together, these designs show that a high score in one dimension is typically obtained at the expense of another, and that no prior tool occupies the center of the space. The Allstruder was developed against a different objective, remaining broadly capable across all five metrics at once and targeting the simplicity and versatility we identified as the primary gaps left by existing DIY designs. On parts designed for 3D printing it matches the Replistruder 5, as both are current designs shaped by extensive experience and focused on reliability and accessibility, and the core Allstruder components have been refined through more than 85 iterations, most aimed at improving success rates during printed part production on standard desktop FFF machines. At $51 per extruder, the Allstruder ranks just behind the Replistruder 3 in affordability while avoiding that design's performance compromises. It does not surpass a purpose-built tool within that tool's own niche, and it will not out-perform a Replistruder 5 paired with a glass syringe on a high-quality motion system, but it delivers strong performance while remaining easier to use and more broadly compatible, which makes it the more practical option across most users and applications. This versatility follows directly from the modular, reconfigurable drive detailed in the Configurability, Mounts, and Adapters section and illustrated in Fig. 5, which supports the syringes shown in Fig. 2E and the use cases shown in Fig. 2F, and allows users to change syringes, substitute stronger components, or extend the mechanical stroke over the course of a biofabrication campaign, from conceptualization through to application and publication. Critically, the mechanical performance retained across all these configurations is governed by proper assembly of the Allstruder components (Fig. 6, Fig. 7, Fig. 8, Fig. 9, Fig. 10, Fig. 11), and is governed by the rigidity of the drive mechanism, quantified in Fig. 13, rather than by any single syringe or printer, so this breadth does not come at the expense of the determinant of extrusion fidelity.

### 2.7 Allstruder mechanism design

**Fig. 3:**
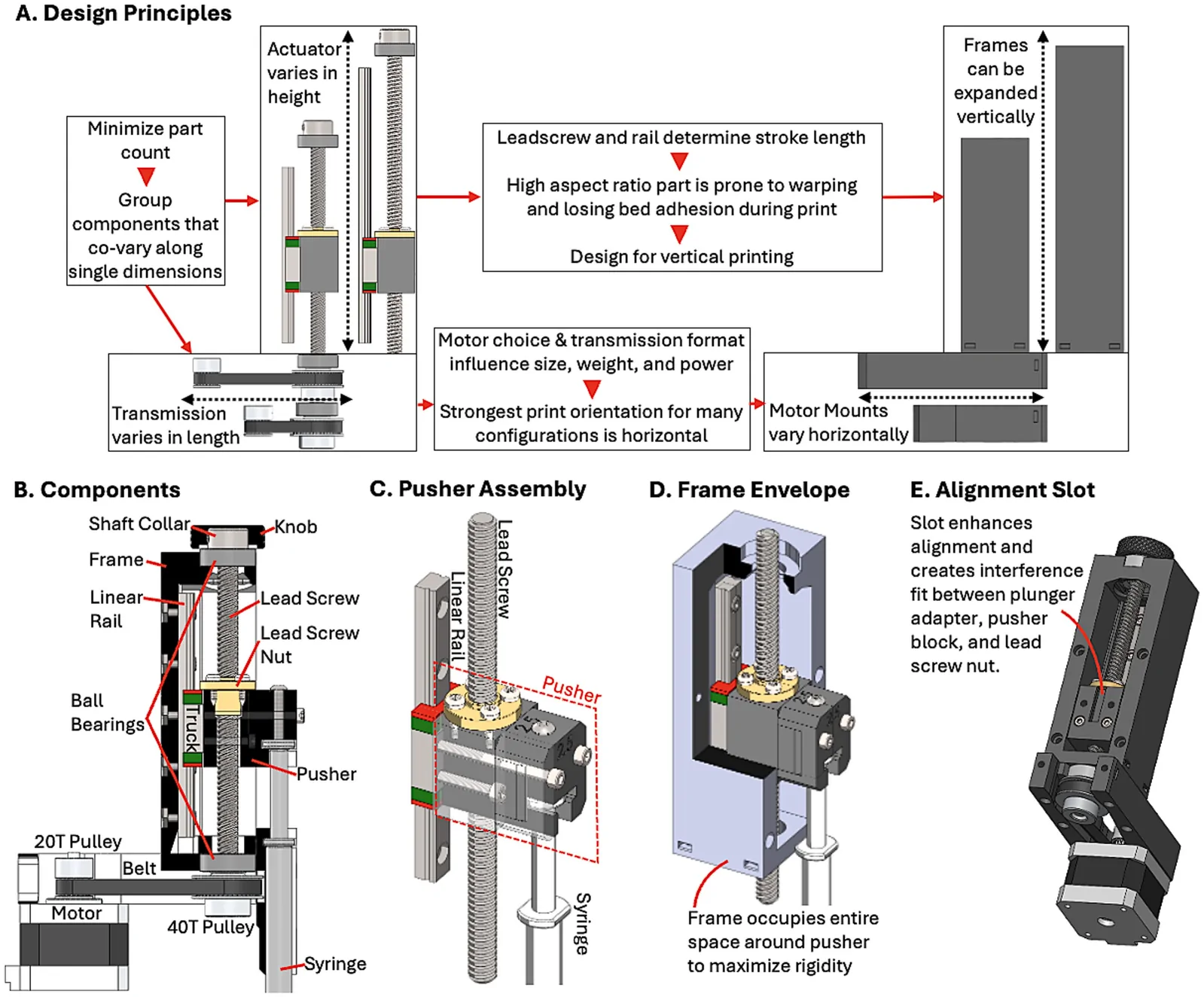
Allstruder Architecture. A) A flowchart demonstrating the reasoning behind the modular design of the Allstruder. B) A cross section showing the components of the Allstruder with a 2.5 mL glass syringe loaded. C) An isometric view rendering of the Allstruder’s pusher assembly, showing the lead screw, linear rail, and syringe attachment points along with transparency to allow visualization of internal assembly hardware. D) An isometric view rendering of the Allstruder actuator with a cutaway section of the frame demonstrating the close fit of the frame to the pusher assembly. E) An isometric view rendering of the Allstruder without syringe and adapters, showing the slot used to align the syringe pusher adapter.

To maximize simplicity, the Allstruder was designed with as few components as possible. The Allstruder also needed to accommodate a wide range of use cases, necessitating a modular and adaptable construction. As illustrated in Fig. 3A, users can easily swap in different motors, linear rails, lead screws, and belted transmissions to configure the system for various mechanical requirements. To support different syringe strokes, the actuator is adjustable in length, and the transmission assembly can be scaled to account for varying motor sizes, belt lengths, and pulley dimensions. The frame, which houses the actuator, is available in standard and extended sizes, offering stroke lengths of 66 mm and 116 mm, respectively. Similarly, the motor mount comes in two sizes: one designed for NEMA 17 motors paired with a 180 mm belt, and a more compact version for NEMA 14 motors using a 120 mm belt.

Allstruder mechanical components, shown in Fig. 2B, were selected for their availability, durability, and performance, making them both reliable and easy to replace. The pusher assembly (Fig. 2C) converts the rotary motion of the lead screw into linear displacement of the syringe plunger. To enhance stiffness, long bolts run the length of the pusher, applying preload across 3D printed parts. The lead screw nut is secured to the pusher block using self-tapping screws, which are easier to install than heat-set inserts and offer stronger, more reliable fastening than conventional bolts threaded into plastic. As seen in Fig. 2D, the surrounding frame fully encloses the pusher, maximizing mechanical support.

Since the pusher is subject to bending and compression forces and must be partially disassembled during syringe adapter changes, proper realignment during reassembly is essential. To address this, the front face of the pusher block includes a keyed slot (Fig. 3E) that mates with a boss on the rear of each syringe adapter. This feature ensures correct assembly every time a syringe adapter is swapped.

### 2.8 Weight distribution and footprint

**Fig. 4:**
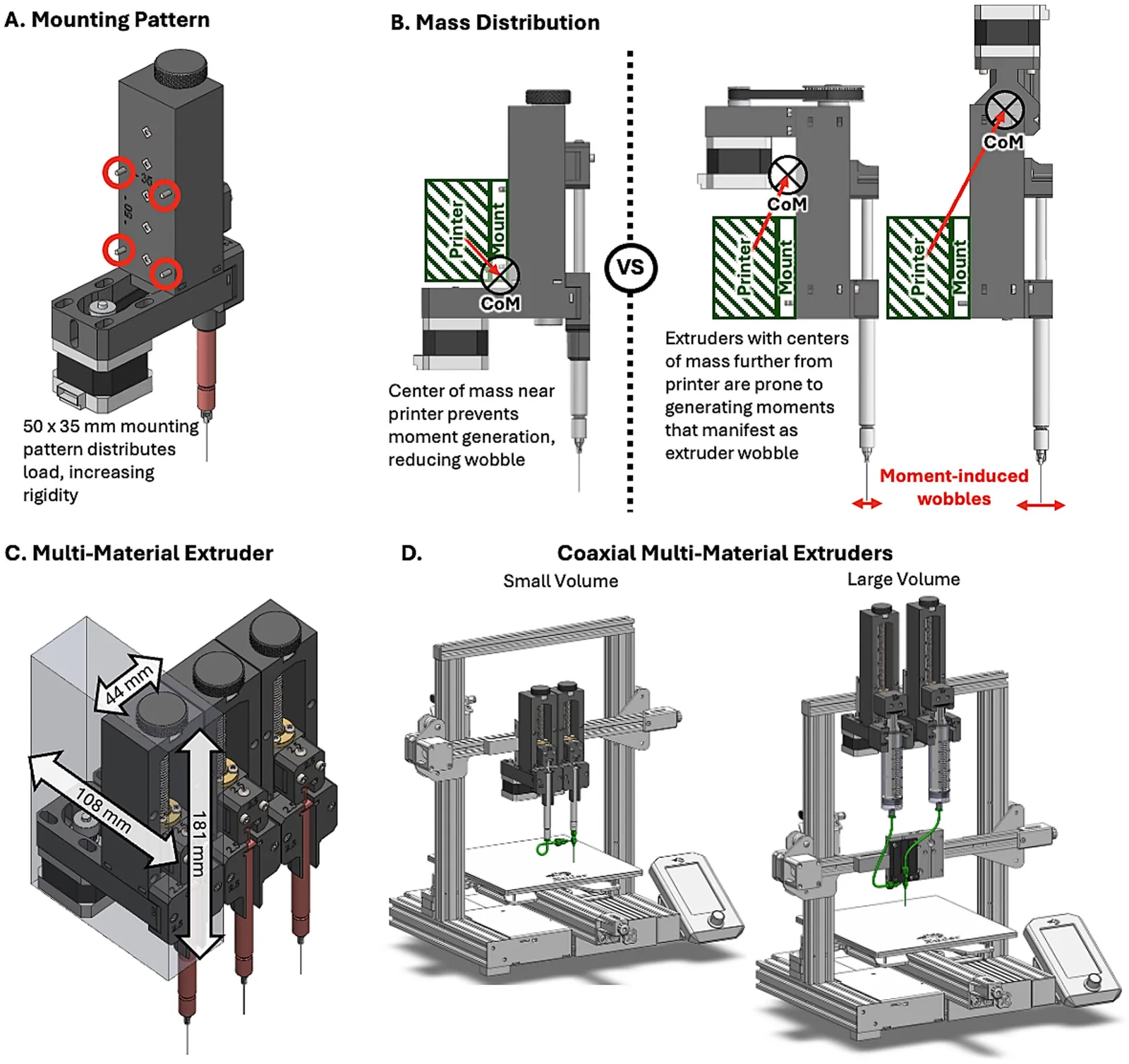
Compact Form Factor Optimized for Responsive Single- and Multi-Material 3D Printing. A) Isometric rear view of the Allstruder, showing the four mounting bolts for sturdy attachment. B) Low center of mass (CoM) enables rapid motion with minimal moment-induced wobble. C) Compact geometry allows dense arraying for multi-material extrusion setups. D) Example coaxial extrusion configurations using multiple Allstruders, rendered on a Creality Ender 3 V2 3D printer, illustrating dual-material feeding through a coaxial spinneret/needle in both small- and large-volume arrangements.

To ensure the Allstruder would perform reliably in high-speed and multi-material 3D printing applications, we paid close attention to its physical footprint and dynamic behavior as a moving payload. Much like a high-performance racing vehicle with a wide wheelbase, the Allstruder uses four widely spaced mounting bolts (Fig. 4A) to secure itself firmly to a printer’s gantry, enhancing stability during rapid movements. Its low center of mass (Fig. 4B) further minimizes vibration and wobble caused by inertia, particularly during sharp directional changes at speed.

**Fig. 5:**
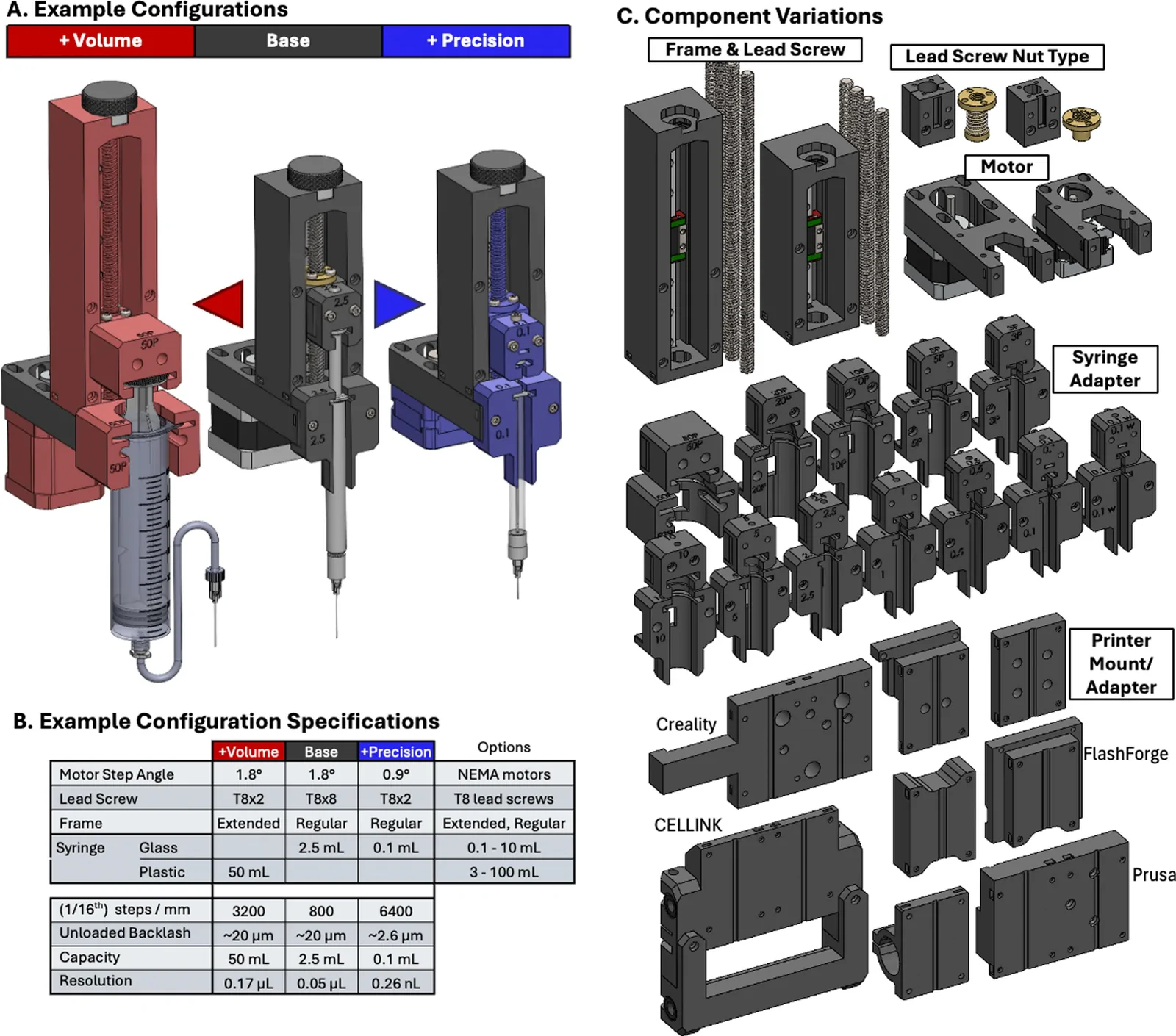
Allstruder Modules Configurable for Diverse Applications Using Many Syringes and Printers. A) Three example configurations of the Allstruder showing the base configuration suggested as a starting point as well as high precision and high-volume optimizations. B) A table explaining the specifications of the three example configurations C) Allstruder project parts.

While compact extruders like the Replistruder 5 (Fig. 2A) are optimized to conserve lateral space in high performance, multi-extruder systems, the Allstruder prioritizes versatility[30]. To support high-torque actuation and broad hardware compatibility, the Allstruder accommodates motors up to NEMA 17 size, and since the motor is the widest component, it determines the overall Allstruder width of approximately 44 mm. (Fig. 4C). For multi-material configurations, the Allstruder supports both direct drive and Bowden-style setups. Multiple Allstruders can be mounted close together to minimize space or further apart, depending on the needs of the user. A potential case for mounting Allstruders further apart might be for printing multiple materials into standard petri dishes, such that a secondary, standby Allstruder’s needle does not collide with the dish while another primary Allstruder is extruding inside it. When configured in a coaxial extrusion system (Fig. 4D), the Allstruder can operate either as a gantry-mounted extruder for small-volume inks or as a frame-mounted pump feeding larger volumes through tubing to the printhead. Many of the printer-specific mounts displayed later in Fig. 5C incorporate mounting points for multiple Allstruders to natively support multi-material setups.

### 2.9 Configurability, mounts, and adapters

To clearly illustrate how the Allstruder can be configured for specific applications, three example setups are shown in Fig. 5A and described in detail in Fig. 5B:

• Base Configuration: Featuring a 2.5 mL glass syringe and matching adapter, this setup offers a balanced starting point for users uncertain about which configuration best suits their needs. It provides a compromise between precision, torque, and ease of use, making it ideal for general-purpose applications. Most users can simply alter this configuration with a different syringe and syringe adapter to meet their needs.
• Large-Volume Bowden Configuration: Designed for high-volume paste extrusion, this example setup includes a 50 mL plastic syringe, matching adapter, extended frame, 150 mm linear rail, 200 mm long 2 mm-pitch lead screw, and a high-torque stepper motor. It is mounted to the printer frame, with extrusion delivered through tubing to a nozzle mounted on the gantry. This Bowden extruder configuration removes mechanical strain from the moving components of the printer while enabling large-volume delivery.
• Extreme Precision Configuration: This setup prioritizes precision and minimal backlash. It includes a 0.1 mL glass syringe, anti-backlash pusher block, 2 mm-pitch lead screw, anti-backlash nut, and a 0.9° stepper motor. Mounted similarly to the base configuration, it delivers highly accurate and repeatable extrusion, ideal for applications requiring fine control, such as when printing precious inks. This configuration can be made even more precise by adding geared motors with more steps per revolution.

The Allstruder platform offers modularity comparable to a LEGO set. Users can mix and match components (as shown in Fig. 5C, and beyond) to create a setup tailored to their specific needs. The growing ecosystem currently includes: 2 frame options, 2 motor mounts, 2 pusher blocks, 12 syringe adapters, 1 Bowden nozzle adapter, 1 syringe heater (in development), and over 10 printer adapters. In general, users are encouraged to begin component selection with the Frame & Lead Screw (Fig. 5C, top left) and proceed clockwise through the options, concluding with the Printer Mount/Adapter.

### 2.10 Broader applications and reuse potential

The Allstruder has been and can be utilized in a variety of ways in- and outside the original user community. Below are a few examples of unique or novel use cases:

• Span materials/methods on a single device. Researchers can move between embedded and FRESH printing and direct ink writing across bioinks, hydrogels, high-viscosity pastes, ceramic and aerogel precursors, edible materials, and conductive inks or silicones, using a single reconfigurable head rather than a separate dedicated printer for each material or technique.
• Build upon the platform rather than re-solving the actuation. Groups developing novel extrusion hardware can treat the Allstruder as a stable, well-characterized base to extend, engineering custom syringes, syringe heaters and chillers, syringe-mounted manifolds, active mixing heads, valved dispensing heads, and other attachments onto it, so that method development focuses on the new component instead of re-solving the underlying functionality.
• Standardize across different host machines. Labs running heterogeneous printer fleets can adopt one extrusion platform across different host machines, reducing the per-printer engineering effort of maintaining bespoke extruders and making protocols and results more portable within and between labs.
• Substitute for a dispenser or syringe pump. Beyond printing, the actuator can serve as a programmable, printer-controlled syringe pump for reagent metering, precise fluid dispensing, or fabricating assay substrates such as immunochromatographic test strips, letting analytical chemistry and lab-automation groups repurpose motion hardware they already own in place of a standalone pump.
• Support educational labs, classes, and projects. The Allstruder can be built for roughly 50 USD from standard hardware and a desktop FFF printer, lowering the barrier to hands-on instruction in additive manufacturing and biofabrication; it has already been used in multiple workshops around the world where commercial syringe-extrusion systems would be cost-prohibitive.
• Serve as a simple, easily modified linear actuator. More broadly, the mechanism can function as a low-cost, reconfigurable linear stage comparable to commercially available motorized actuators, offering researchers an openly documented and readily-modified alternative for general precision-motion tasks beyond extrusion.

## 3 Design files summary

**Table:** 

Design File	File Type	Open Source License	Location of the file
Frame	STP, STL	CERN OHL v2	https://doi.org/10.5281/zenodo.15670349
Frame_Extended	STP, STL	CERN OHL v2	https://doi.org/10.5281/zenodo.15670349
Pusher_Block	STP, STL	CERN OHL v2	https://doi.org/10.5281/zenodo.15670349
Pusher_Block_Anti-Backlash_Top	STP, STL	CERN OHL v2	https://doi.org/10.5281/zenodo.15670349
Pusher_Block_Anti-Backlash_Bottom	STP, STL	CERN OHL v2	https://doi.org/10.5281/zenodo.15670349
Knob	STP, STL	CERN OHL v2	https://doi.org/10.5281/zenodo.15670349
Motor_Mount	STP, STL	CERN OHL v2	https://doi.org/10.5281/zenodo.15670349
Motor_Mount_NEMA_14_120mm_Belt	STP, STL	CERN OHL v2	https://doi.org/10.5281/zenodo.15670349
Syringe_Pusher	STP, STL	CERN OHL v2	https://doi.org/10.5281/zenodo.15670349
Syringe_Holder	STP, STL	CERN OHL v2	https://doi.org/10.5281/zenodo.15670349
Needle_Mount_Bowden_Extruder	STP, STL	CERN OHL v2	https://doi.org/10.5281/zenodo.15670349
Allstruder_Mount_Printer	STP, STL	CERN OHL v2	https://doi.org/10.5281/zenodo.15670349

Frame: This is the largest single part and provides a housing for the linear actuator components such as the lead screw, linear rail, pusher assembly, etc.

Frame_Extended: A 50 mm taller variant of the frame, intended for use with 200 mm leadscrews and 150 mm linear rails.

Pusher_Block: This part serves as the mobile element in the actuator, connecting the lead screw nut and the linear rail truck to actuate the syringe pusher adapter.

Pusher_Block_Anti-Backlash_Top: First half of a variant of the pusher block designed to accommodate anti-backlash nuts. This is for extreme precision and low thrust applications with microliter volume syringes.

Pusher_Block_Anti-Backlash_Bottom: Second half of a variant of the pusher block designed to accommodate anti-backlash nuts. This is for extreme precision and low thrust applications with microliter volume syringes.

Knob: A knurled attachment that enables manual actuation of the Allstruder’s lead screw.

Motor_Mount: This is the housing for the transmission, it serves as the mounting point for syringe holder adapters, and it connects the actuator to any NEMA 17 motor using a 180 mm belt.

Motor_Mount_NEMA_14_120mm_Belt: A shorter variant of the motor mount intended to connect any NEMA 14 motor to the actuator using a shorter 120 mm belt.

2.5_Hamilton_Pusher: The top half of the syringe adapter for a Hamilton 2.5 mL glass syringe. This part actuates the syringe plunger and connects to the Allstruder’s pusher block. This file is one of the many syringe adapters for the syringes listed in Fig. 2E.

2.5_Hamilton_Holder: The bottom half of the syringe adapter for a Hamilton 2.5 mL glass syringe. This part holds the barrel of the syringe stationary and connects to the Allstruder’s motor mount. This file is one of the many syringe adapters for the syringes listed in Fig. 2E.

Needle_Mount_Bowden_Extruder: This part is for applications where materials are extruded from nozzles connected by tubing to remote Allstruders mounted to a printer’s frame, such as when a large volume of material would make an extruder too heavy to reasonably operate on a mobile gantry. This part allows a needle with tubing to connect in place of an Allstruder on a printer’s extruder gantry, using the same printer mount/adapter as the Allstruder. This part is intended for use with stainless steel needles and coaxial needles such as those manufactured by ramé-hart instrument co.

## 4 Bill of materials summary

**Table:** 

Component	# Needed	SKU	#/SKU	Source	USD/SKU	USD/Allstruder
100 mm MGN9C Linear Rail Kit	1	B07WNXP2K5	1	Amazon	$7.89	$7.89
150 mm Tr8X8 Leadscrew, Nut	1	B07C8P1DWX	1	Amazon	$12.99	$12.99
608 ZZ/RS Bearings	2	B07R7PR72H	10	Amazon	$7.99	$1.60
20 T 2GT Pulley 5 mm Bore	1	B077GNZK3J	5	Amazon	$6.99	$1.40
40 T 2GT Pulley 8 mm Bore	1	B09JSMRZH9	2	Amazon	$4.99	$2.50
Nema 17 Motor 63.74 oz-in	1	B07LF898KN	1	Amazon	$11.99	$11.99
180 mm 2GT Belt	1	B014QLQBQ0	10	Amazon	$9.99	$1.00
8 mm Shaft Collar	1	B0BZ7C2X2W	12	Amazon	$8.99	$0.75
Thin M8 Washer	1	B0C3TV2QDX	20	Amazon	$5.89	$0.29
Thin Square M3 Nuts	1	B0D6SPHN8G	50	Amazon	$11.49	$0.23
8 mm M4 Bolt	1	B0BHQZHSLQ	100	Amazon	$8.38	$0.08
10 mm M3 Bolts	15	B08R3GVJVC	100	Amazon	$8.26	$1.24
20 mm M3 Bolts	2	B089KT7ZNW	100	Amazon	$8.96	$0.18
40 mm M3 Bolts	2	B0BGM73YG8	100	Amazon	$8.22	$0.16
45 mm M3 Bolts	4	B089KRYJ9Q	50	Amazon	$8.42	$0.67
8 mm M3 Self Tapping Screw	4	B09CPHWX7P	625	Amazon	$10.99	$0.07
PETG Plastic Filament	∼200 g	B07PGYHYV8	1 kg	Amazon	$15.99	$3.20
Cost (USD)					$158.42	$46.24

The Allstruder is built using hardware listed in the Bill of Materials along with 3D printed components, and it can be divided into four main sections, as shown in Fig. 1A–D. Before assembly, all printable parts and required hardware should be collected. Assembly primarily occurs in a single plane, as illustrated in the exploded view in Fig. 1H. Once assembled, the Allstruder can function as a standalone syringe pump or as an extruder mounted to a printer, shown in Fig. 1I.

## 5 Build instructions

**Fig. 6:**
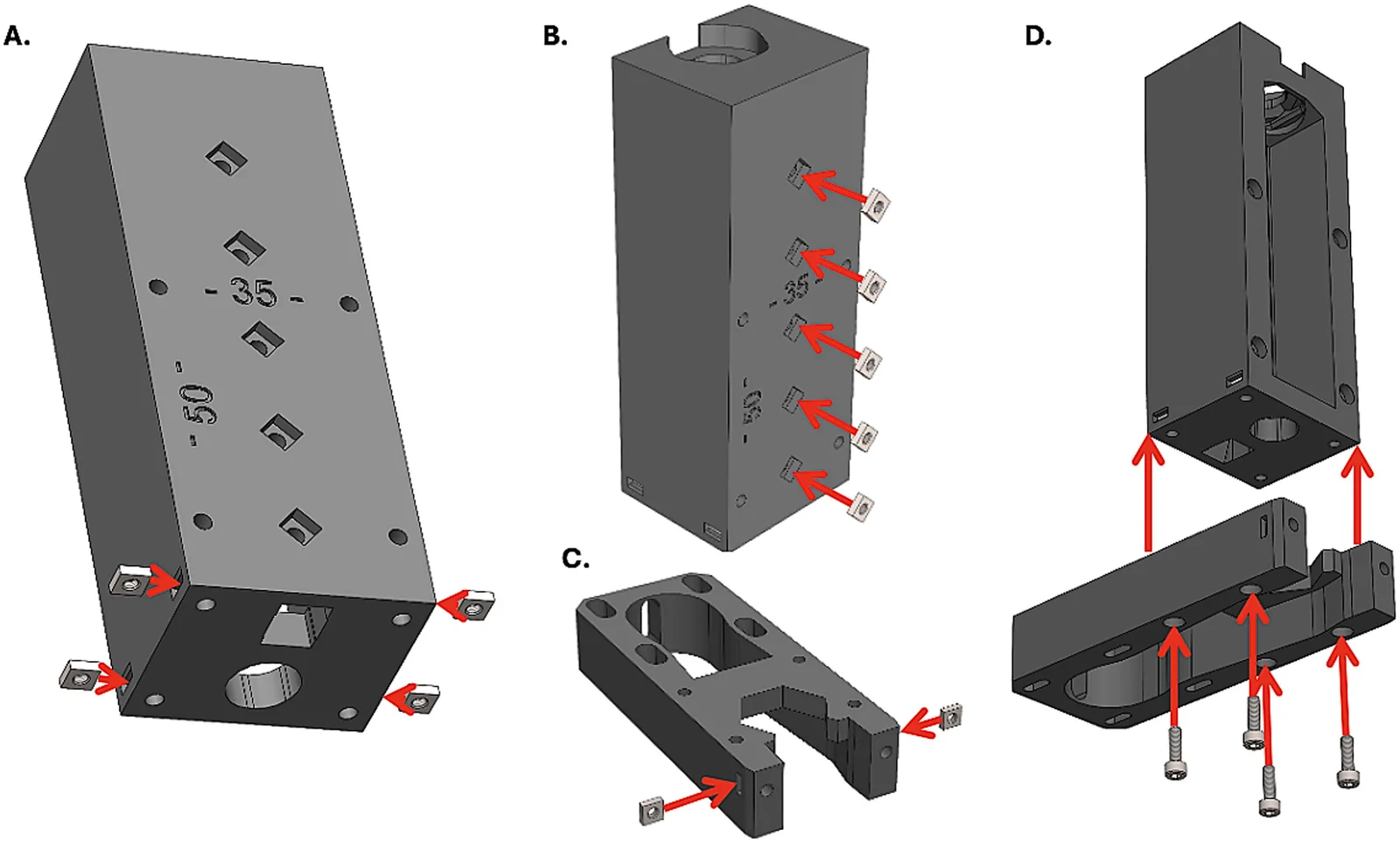
Assembly Steps 1 – 4. A) Step 1 − Fully insert 4x thin square M3 nuts into the slots at the base of the frame. B) Step 2 – Fully insert 5x thin square M3 nuts into the diamond-shaped recesses in the back of the frame. C) Step 3 – Fully insert 2x thin square M3 nuts into the slots at the front of the motor mount. D) Step 4 – Attach the motor mount to the frame by inserting and tightening (to finger tightness) with 4x M3 10 mm bolts.

**Fig. 7:**
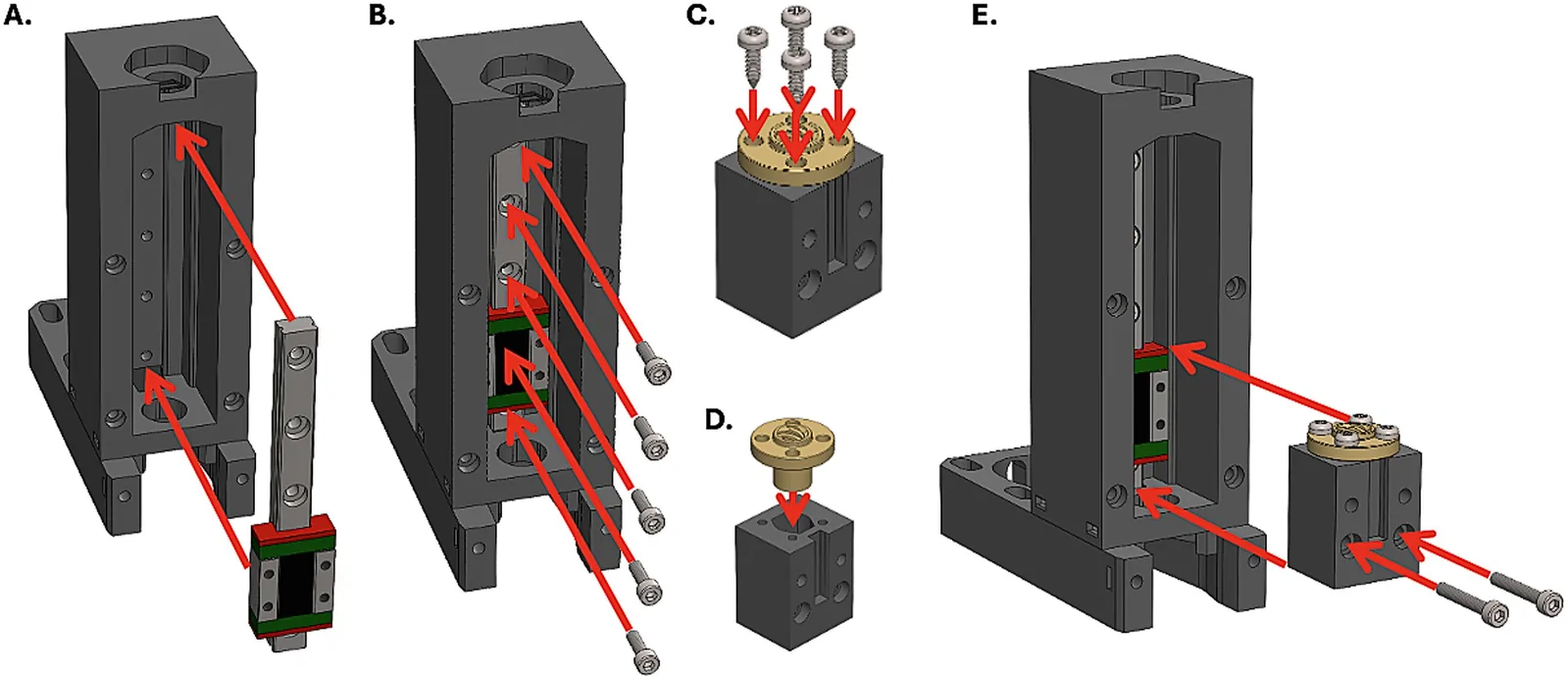
Assembly Steps 5 – 9. A) Step 5 – Carefully insert the linear rail into the frame, ensuring it lines up with the groove in the rear of the front cavity. B) Step 6 – Attach the rail to the frame by installing and tightening (to finger tightness) 5x M3 10 mm bolts through the holes in the rail, moving the truck out of the way to reach vacant holes. C) Step 7 – Fully insert the lead screw nut into the pusher block as shown. D) Step 8 – Attach the lead screw nut to the pusher block using 4x M3 8 mm self-tapping screws. It may be necessary to rotate the lead screw nut to align its holes with those of the pusher block before installing the screws. E) Step 9 – Attach the pusher block to the linear rail truck by installing and tightening (to finger tightness) 2x M3 20 mm bolts through the lower holes in the pusher block, making sure the pusher block sits directly over the linear rail truck.

**Fig. 8:**
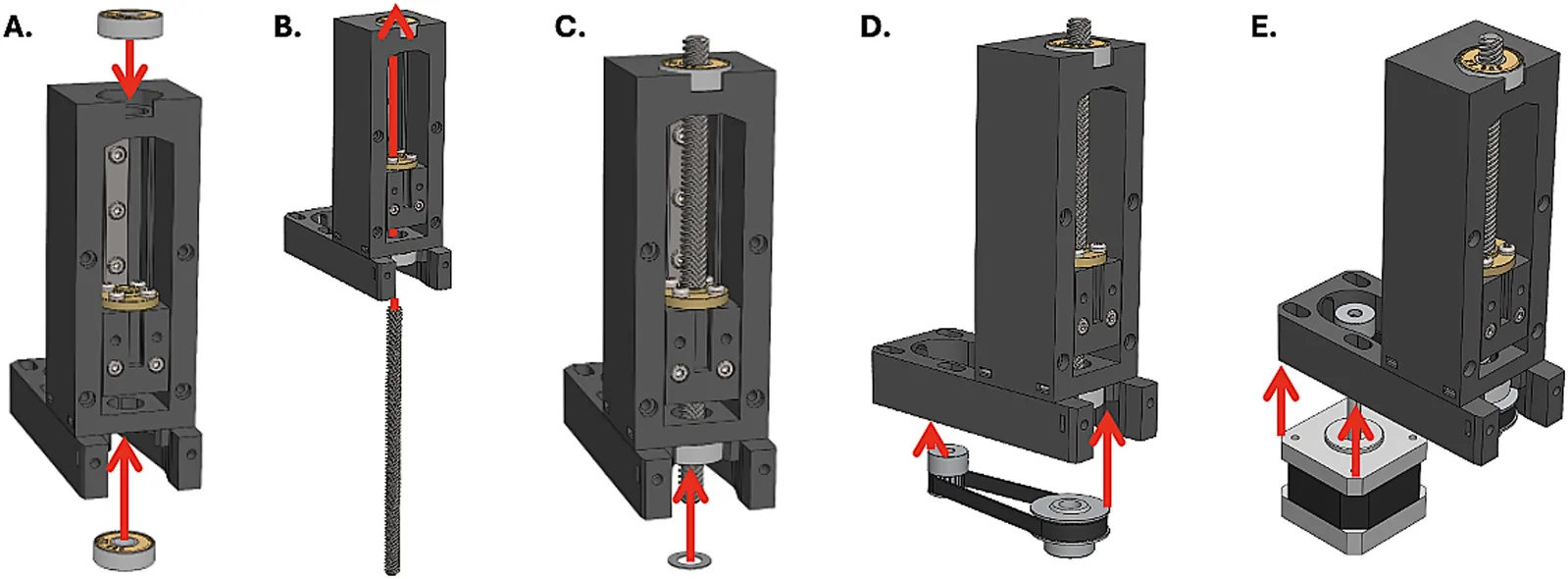
Assembly Steps 10 – 14. A) Step 10 – Fully insert 2x 608 ball bearings, one into the hexagonal recess at the top of the frame, and the other into the hexagonal recess on the bottom of the frame, within the motor mount. B) Step 11 – Insert the lead screw through the bottom ball bearing, pusher block, lead screw nut, and finally the top ball bearing. Stop when the lead screw has about 6 mm sticking above the top ball bearing. In case the lead screw feels like it is stuck or experiencing excess friction, temporarily loosen the bolts from steps 4 and 9 and reevaluate alignment. If doing so fixes the friction, finish advancing the lead screw then gently tighten the bolts and check for friction. If the friction is gone, proceed; if not, start over while checking for misalignment or part defects. C) Step 12 – Place a thin M8 washer onto the bottom of the lead screw. D) Step 13 – Install the 20 T and 40 T pulleys and belt into the motor mount, onto the bottom of the lead screw. The 20 T pulley should be oriented with the teeth down and the 40 T with the teeth up. With everything installed and ensuring the lead screw is sticking up at least 6 mm above the top ball bearing (the lead screw will end up being flush on both ends after full assembly), tighten the set screws on the 40 T pulley to finger tightness. Be careful not to over-tighten the set screws. E) Step 14 – Place the motor by guiding the shaft into the 20 T pulley and then aligning the motor bolt holes with the motor mount holes.

**Fig. 9:**
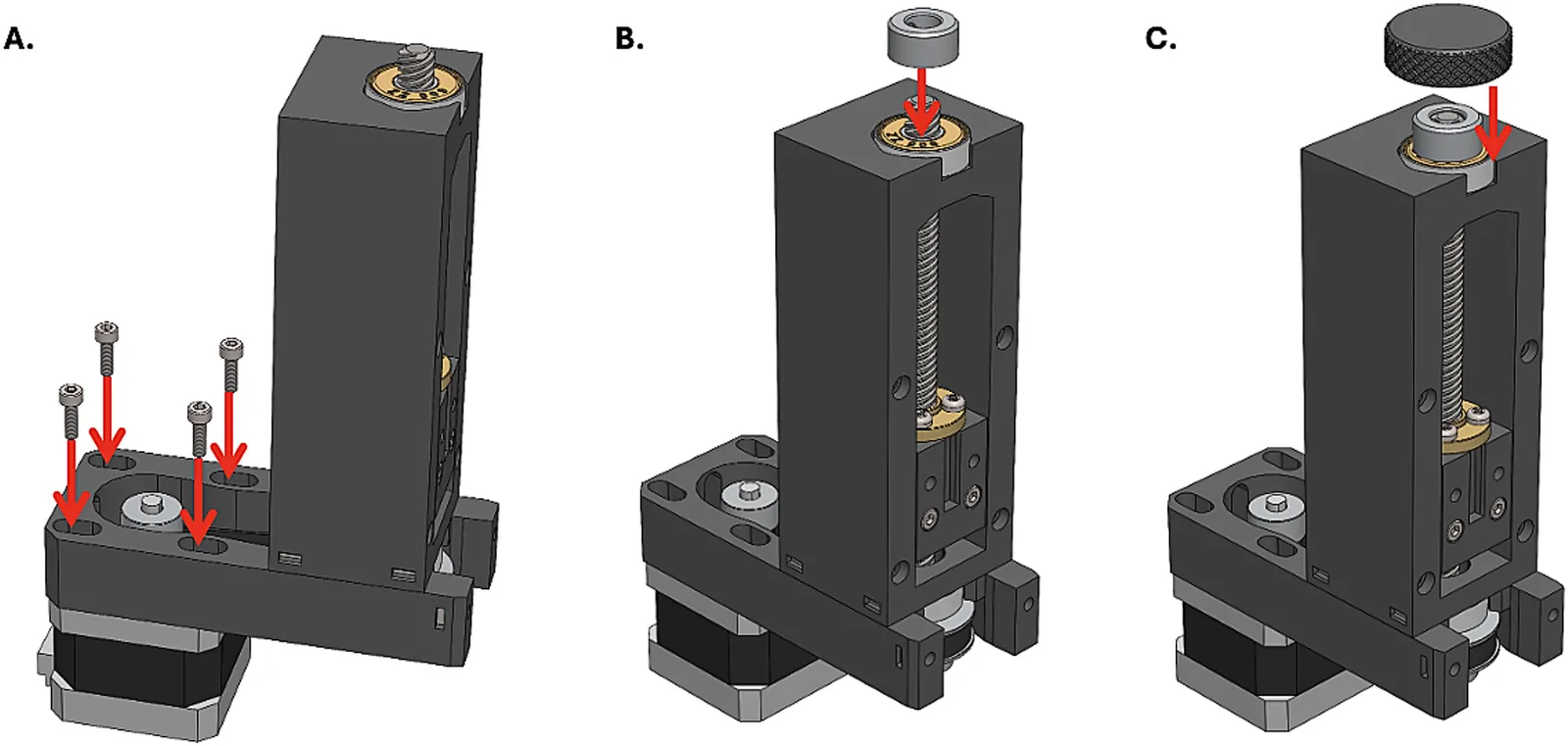
Assembly Steps 15 – 17. A) Step 15 – Attach the motor to the motor mount by inserting and tightening (to finger tightness) 4x M3 10 mm bolts. Loosen the bolts slightly. Pull the motor back to tighten the belt, then re-tighten the 4x M3 10 mm bolts. Using the slot in the back of the motor mount, tighten the set screws in the 20 T pulley (to finger tightness). Be careful not to over-tighten these screws and if the motor shaft has a flat side, tighten one set screw against it to prevent rotation. B) Step 16 – Install the shaft collar (with M4 8 mm bolt) onto the top of the lead screw, tightening the M4 8 mm bolt to finger tightness while ensuring no slack exists along the lead screw axis. After installation, it should be impossible to jostle the lead screw along its axis. If the lead screw still moves back and forth, loosen and re-install the collar, ensuring all slack is removed during re-installation by applying compression to the shaft collar and bottom pulley while tightening. C) Step 17 – Push the knob onto the collar with the M4 8 mm bolt sticking out, ensuring the cutout on the knob lines up with the M4 8 mm bolt. The Allstruder extruder is complete and functional. The user may proceed to step 22 (installing on printer) or step 18 (installing a syringe).

**Fig. 10:**
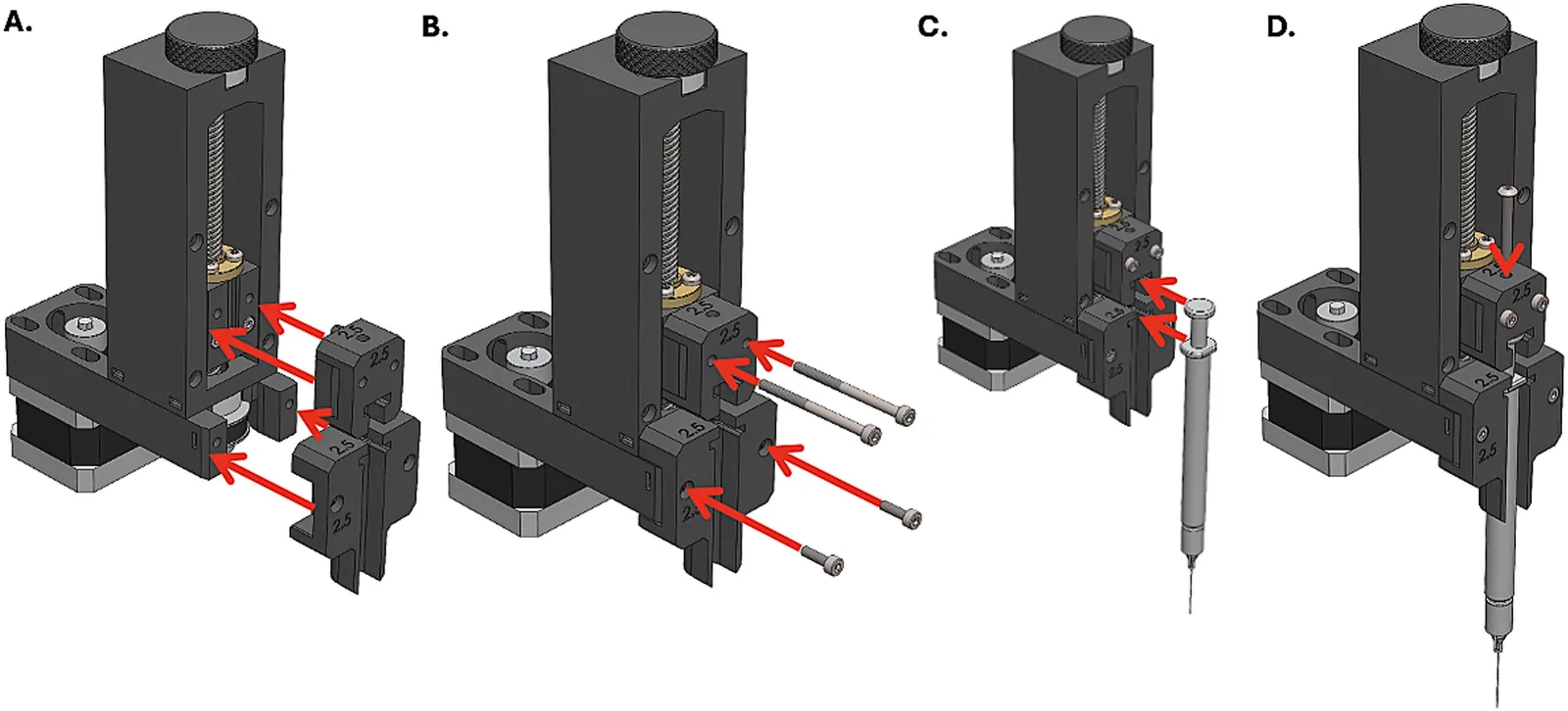
Assembly Steps 18 – 21. A) Step 18 – Attach a syringe adapter set onto the front of the Allstruder. The syringe pusher adapter fits into the pusher block with a boss that fits into the pusher’s slot, and the syringe holder adapter fits onto the two bosses of the motor mount. B) Step 19 – The syringe adapters are fully attached to the Allstruder by bolts. The syringe pusher adapter is attached to the pusher block by 2x M3 40 mm bolts, and the syringe holder adapter is attached to the motor mount by 2x M3 10 mm bolts. Each bolt is tightened to finger tightness. C) Step 20 – The syringe can now be placed in the Allstruder. The Allstruder can be manually adjusted by the top knob to ensure the syringe adapters are at the correct spacing to accommodate the syringe. D) Step 21 – For Hamilton glass syringes, a 6–32 x 25 mm thumb screw or bolt is installed and tightened (to finger tightness) through the top of the syringe pusher adapter to guarantee maximum grip on the syringe plunger.

**Fig. 11:**
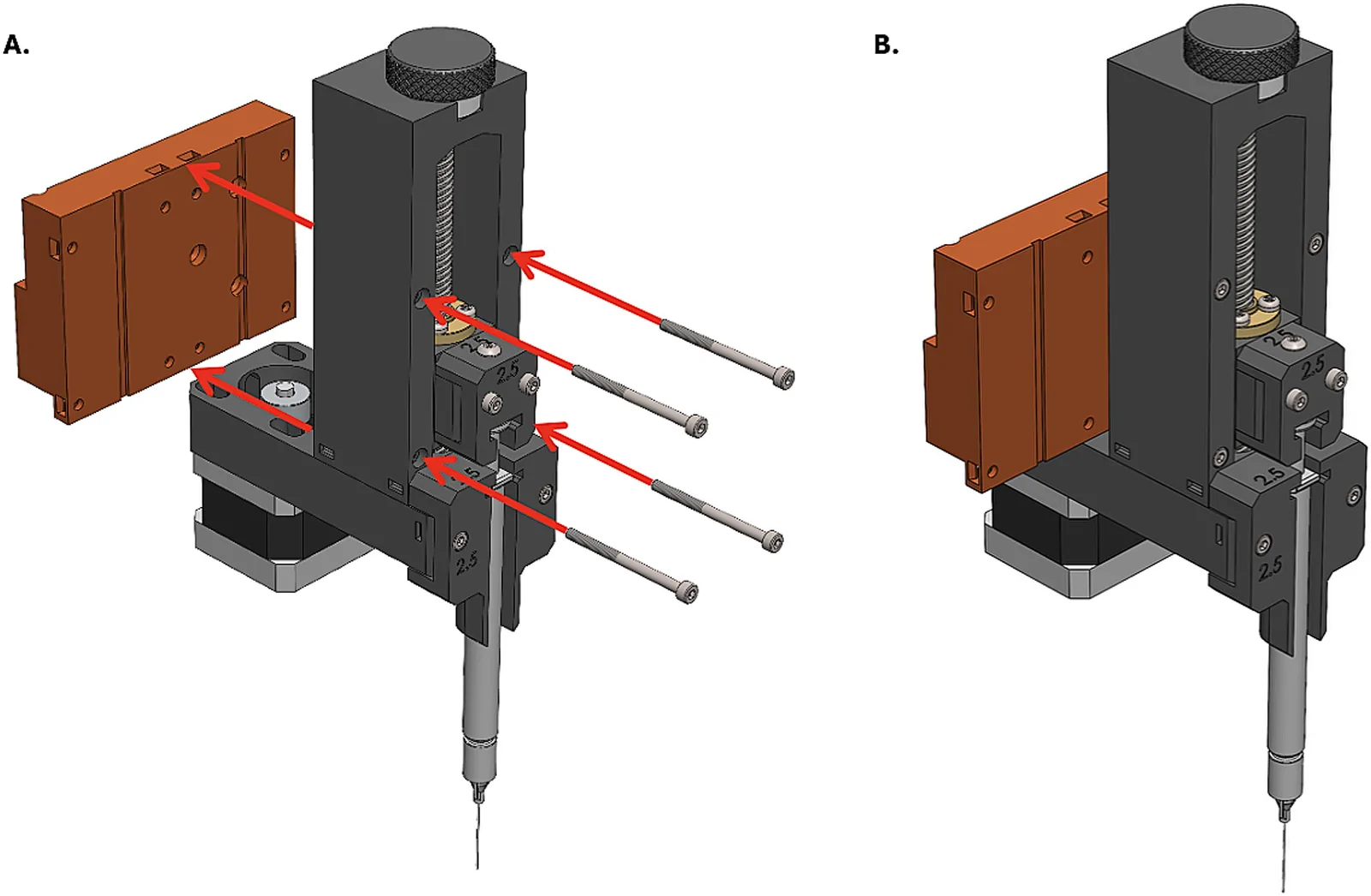
Assembly Steps 22 – 23. A) Step 22 – A completed Allstruder is attached to a printer by a mount/adapter (example Prusa MK4 mount shown in orange) that accepts 4 bolts through the holes on the front of the Allstruder frame. Each printer is a little different, but every Allstruder mount uses the same mounting pattern of 4 holes spaced in a 35 x 50 mm rectangular pattern. To attach, use 4x M3 40 mm bolts and tighten the bolts to finger tightness. B) Step 23 – The Allstruder is installed on the printer and is ready to be connected like any stepper-based extruder. It is possible to use any NEMA 17/14 motor with the Allstruder, and it is expected that most users will use the default extruder motor from their printer or one of the recommended motors in the Bill of Materials. Assuming the user used the extruder motor already on their printer, connecting the Allstruder is no different from the way the extruder motor was originally wired. (For interpretation of the references to colour in this figure legend, the reader is referred to the web version of this article.)

A build video explaining the (Fig. 6, Fig. 7, Fig. 8, Fig. 9, Fig. 10, Fig. 11) assembly is the recommended method for following along and assembling the Allstruder, and it can be accessed through the supplemental information for this paper and alternatively through the Allstruder GitHub. To build the Allstruder, a user will first need to gather the hardware listed in the BOM and print the 3D printable components described in Fig. 1B-E. The printable components of the Allstruder were designed to be printed with PETG, and success has also been achieved with PCTG and some photopolymer resins such as Formlabs Clear Resins. PLA is not recommended for long-term use because of its tendency to creep under load, and ABS, ASA, and PC are only recommended with a 3D printer that can mitigate part deformation during printing. Allstruder part files are available as STL, 3MF, and STP, and each file is in the correct orientation for FFF printing. Project files with the Allstruder parts pre-oriented with appropriate settings for PETG on a Prusa MK4 are available through the supplemental information for this paper and alternatively through the Allstruder GitHub.

## 6 Operation instructions

The Allstruder operates similarly to other DIY syringe pump extruders. The general procedure for setup and use is as follows:

### 6.1 Verify mechanical function and mount the Allstruder

After assembling the Allstruder, the next step is checking that the assembly operates as expected. Rotation of the leadscrew should be possible using minimal manual force, and there should be no axial slop in the leadscrew itself. If rotation of the leadscrew requires a great deal of torque, check that the leadscrew nut is correctly installed in the pusher. If the leadscrew can be moved along its axis, repeat step 16 of the assembly, ensuring that the leadscrew is pushed toward the collar nut while it is being installed around the top of the leadscrew, so as to eliminate any axial slop. The leadscrew should be unable to wiggle but easy to rotate. If repeating step 16 multiple times does not resolve axial slop, it may be necessary to rotate the collar ¼ turn before re-tightening it onto the leadscrew to prevent the collar’s grub screw (if it has one) from pushing the collar up. Other potential problems to watch for include loose pulleys – double check that no pulley can move along leadscrew/motor shaft. If a pulley is loose, tighten the pulley screws until the pulley is securely attached.

Following mechanical function verification, it is time to install the Allstruder on a 3D printer. Most desktop 3D printers using open-source firmware (e.g., Marlin, RepRapFirmware, Klipper) are compatible with the Allstruder due to their use of standard stepper motors. Closed-firmware systems are not recommended, though their motion hardware can often be repurposed by replacing the controller with an open-source board, such as a Duet. Mounting an Allstruder to a suitable printer requires fabricating and installing a compatible mount. For many common printer models, we have provided 3D-printable mounts that are available in the supplementary materials of this paper or on the Allstruder GitHub repository. For printers without an existing mount, users may need to remove the original thermoplastic extruder, carefully measure the extruder gantry, and prototype a custom mount before installing it in place of the original component.

### 6.2 Connect the syringe pump to the 3D printer or motor controller

Once mounted, the Allstruder must be electrically connected to the printer or motor controller. This usually involves attaching (usually plugging into; soldering is rare) a 4-wire cable from the Allstruder’s stepper motor to the controller, typically a standard 3D printer motherboard. After the connection is made, the motor controller must be properly configured to operate the Allstruder. For firmware on most 3D printers, the most critical setting is the steps/mm value used for extrusion. For a standard Allstruder equipped with a TR 8x8 lead screw, the steps/mm can be calculated as follows in equations (1) (Eq. (1)) and 2 (Eq. (2)).:

(1) $$\frac{3200\;\left(micro\right)steps}{1\;fullmotorrevolution}*\frac{40\;leadscrewpulleyteeth}{20\;motorpulleyteeth}*\frac{1\;leadscrewrevolution}{8\;mmpushertravel}=800\frac{\mathrm{steps}}{\mathrm{mmtravel}}$$

For Klipper firmware-based 3D printers, the equivalent value used to control the extruder stepper movement is rotation distance and is calculated as follows:

(2) $$\left(\frac{3200\;\left(micro\right)steps}{1\;fullmotorrevolution}\right)*\frac{1\;mmtravel}{800\;steps}=4\frac{\mathrm{mmtravel}}{\mathrm{fullmotorrevolution}}$$

### 6.3 Load the syringe with material

Ideally, the syringe is loaded with material in a manner that avoids trapping or generating bubbles, since bubbles always negatively impact the predictability of extrusion. There are a few places where bubbles can become trapped within the system: i) within the material, ii) a dead volume space such as that found within a needle hub, and iii) against a surface such as a black rubber plunger. Alternatively, bubbles can be generated by ink materials that were previously mixed and refrigerated but have been left out at ambient temperature. Warming causes dissolved gasses to nucleate within the syringe/needle, and as such, printing materials should be degassed only after warming to room temperature.

To load a syringe and needle without bubbles, there are many methods, and the following steps should cover most materials in most plastic syringes:

i) Soak a needle (or several) in a very low concentration of a surfactant such as 0.1% w/v Pluronic F127
ii) Remove the plunger from the syringe by pulling it out the back
iii) Cap the bottom of the syringe barrel (which is most often a luer fitting)
iv) Fill the syringe from the back/top with the material to be printed
v) Centrifuge the syringe barrel/material to remove all bubbles (load syringe with cap side down and open back facing up and carefully balance centrifuge). For glass or uncommon syringes such as precision or large volume models, it is advisable to design and print centrifuge syringe collars/holders for use in this step.
vi) Insert the syringe plunger along with a very thin wire down into the barrel to allow all air/bubbles to escape past the plunger in the gap the wire makes and keep inserting until the plunger contacts the top of the fluid material
vii) Remove thin wire, restoring the seal of the plunger against the inside of the syringe barrel
viii) Rinse a surfactant-washed needle (or several) with distilled water
ix) Remove cap from syringe barrel and attach a needle to syringe barrel
x) Carefully actuate syringe plunger to extrude material into needle hub, ensuring it wets all surfaces within needle hub and does not trap any air. This is particularly challenging with highly viscous and yield-stress fluids. Tapping the syringe can help dislodge bubbles and allow them to be pushed out the needle tip. If a bubble is formed, discard needle and get a clean one or manually remove trapped bubble with another syringe/needle acting as a vacuum.
xi) Success − syringe and needle are now loaded without bubbles.

### 6.4 Prepare G-code or other appropriate machine instructions

There are many effective ways to set up G-code for printers using one or more experimental extruders, making it impossible to cover every possible configuration here. Likewise, G-code can be structured in numerous ways depending on the material and printing approach, and many different software tools can generate valid results. As one example, the following settings demonstrate how PrusaSlicer can be used to generate G-code for printing a single material with an Allstruder-equipped printer configured to act purely as a G-code interpreter. This process does not require specialized pre- and post-processing scripts (e.g., homing, calibration) typically used in thermoplastic printing. For users running Klipper firmware, executing Allstruder-specific G-code may require disabling or bypassing macros such as START_PRINT and END_PRINT, which are normally associated with thermoplastic workflows. The extremely simplified example below was developed to enable direct ink writing of mayonnaise − that is, printing mayonnaise in air using a 5 mL plastic syringe with a 27 gauge needle on a Creality K1 SE fitted with an Allstruder. The corresponding PrusaSlicer profile is available in the Allstruder GitHub repository.

**Table:** 

Sample FRESH Printing Profile Parameters	Sample Parameter Values
Layer Height:	0.1
Extrusion Width:	0.25
Number of Perimeters:	2
Infill %:	100%
Extrusion Speed:	23 mm/s
Travel Speed:	150 mm/s
Retraction/Deretraction Distance:	0.6 mm
Retraction/Deretraction Speed:	0.5 mm/s
Z-Hop During Travel:	0.25 mm
Start G-Code:	SET_KINEMATIC_POSITION X = 110 Y = 110 Z = 0 E = 0
End G-Code:	CANCEL_PRINT

### 6.5 Setup the printer for the print

Load the prepared syringe into the Allstruder. The Allstruder is designed for front-loading, allowing the syringe to be inserted with a simple press fit. If the syringe feels loose, the adapter may need to be modified, or thin layers of tape can be applied to the syringe barrel to create a snug, stable fit.

Next, prepare the print container, with or without support material:

• For printing in air: Secure the surface that will serve as the print base.
• For embedded / FRESH printing: Secure the container holding the support material and ensure the needle can enter the bath without obstruction. Verify that the syringe and needle hub will not collide with the sides of the container during movement.

Before starting the print, confirm that the Allstruder can extrude properly. Clogging or lack of extrusion can result from dried material on the needle tip, a disconnected motor cable, or solidified material inside the syringe. A simple test is to command a small extrusion via the printer’s control interface and visually confirm continuous material flow from the needle. Then wipe the needle clean before proceeding.

Finally, position the needle or nozzle tip at the correct starting location:

• For printing in air: Align the needle tip just above the print surface (e.g., the base of a petri dish).
• For embedded / FRESH printing: Position the needle at the desired depth within the support material, ensuring it is fully submerged and clear of nearby walls or obstructions.

### 6.6 Initiate and monitor the print process

Once the print is initiated, there are several factors to monitor during operation. Many printer firmwares and default slicing profiles include start and end scripts designed for conventional FFF/FDM printing workflows. These profiles handle homing, heating, and cooldown sequences automatically, and can be defined for specific materials. When using an Allstruder, these scripts can interfere with the desired behavior. Unintended motions, fans starting, or automatic heating of the thermoplastic extruder or build plate are clear signs that such scripts are still active and should be disabled. During longer prints, particularly in dry environments, users should also be aware of potential dehydration of either the printed material itself or, in the case of embedded printing (FRESH), the support bath. Air bubbles within the syringe or needle can lead to intermittent flow or cause retraction and priming moves to become unresponsive. In addition, small accumulations of material around the needle tip may interfere with deposition if they contact previously extruded material.

## 7 Validation and characterization

The Allstruder was tested across a diverse network of labs, each with unique materials, methods, printers, and user experience levels. By distributing the design and build instructions broadly and collecting iterative feedback, we identified and resolved adoption-limiting issues, resulting in a robust, user-friendly tool capable of reproducible printing across a wide range of settings. This process validated the Allstruder’s performance with hydrogels, pastes, and other viscous or non-Newtonian materials, while also confirming its compatibility with varied printer architectures and syringe extrusion workflows.

**Fig. 12:**
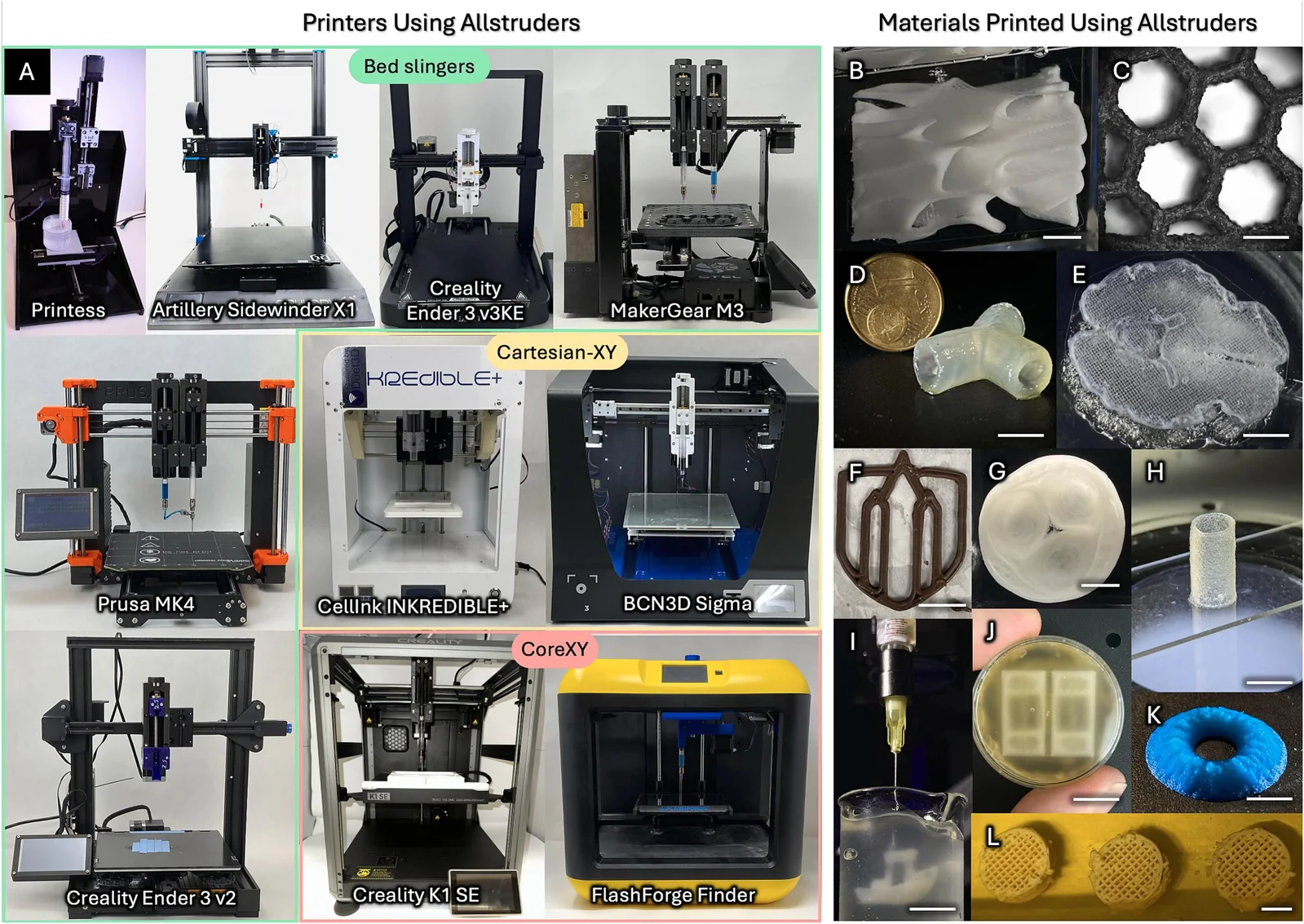
Allstruder Is Platform Agnostic to Printer Brand and Motion Configuration Style. A) Examples of printers modified with Allstruders, some in multi-material configurations. B) A soy protein scaffold produced using FRESH/embedded printing. Scale bar = 4 mm. C) A granular hydrogel scaffold produced using direct ink writing. Scale bar = 2 mm. D) A novel protein hydrogel scaffold produced using FRESH/embedded printing. Scale bar = 8 mm. E) A protein brain model produced using FRESH/embedded printing. Scale bar = 2 mm. F) A chocolate Southwestern University logo produced using direct ink writing. Scale bar = 20 mm. G) A soy protein heart valve model produced using FRESH/embedded printing. Scale bar = 6 mm. H) A granular hydrogel tube produced using direct ink writing. Scale bar = 10 mm. I) A soy protein 3DBenchy being printed using FRESH/embedded printing. Scale bar = 10 mm. J) Two identical collagen microfluidic chips produced using FRESH/embedded printing. Scale bar = 10 mm. K) A protein model of a human iris printed using FRESH/embedded printing. Scale bar = 5 mm. L) Three aerogel precursor disks produced using FRESH/embedded printing. Scale bar = 5 mm.

Integrating the Allstruder into each research setup required understanding the specific needs of the user. These needs can vary but normally include their printer model, printing material, target object, and syringe requirements. For example, the Printess, a low-cost, open-source, small-format bioprinter designed by the Skylar-Scott lab is a highly modular extrusion-based 3D printing platform that was made compatible with the Allstruder by designing a custom mount tailored to its dimensions[24]. This adaptation enabled the Printess to benefit from the Allstruder’s high torque and large-volume extrusion capabilities, while continuing to provide affordability. The modified Printess is shown as the first printer in Fig. 12A. Across the remainder of Fig. 12A, a wide variety of printers have been adapted to use the Allstruder, ranging from affordable Creality Ender 3 V2s to a CellInk INKREDIBLE. These integrations have led to the creation of numerous novel (largely unpublished) materials and printer configurations, examples of which are shown in Fig. 12C. Many researchers have contributed to the development of the Allstruder by offering critical feedback on its functionality and helping to expand the library of components, including new printer mounts and syringe adapters.

**Fig. 13:**
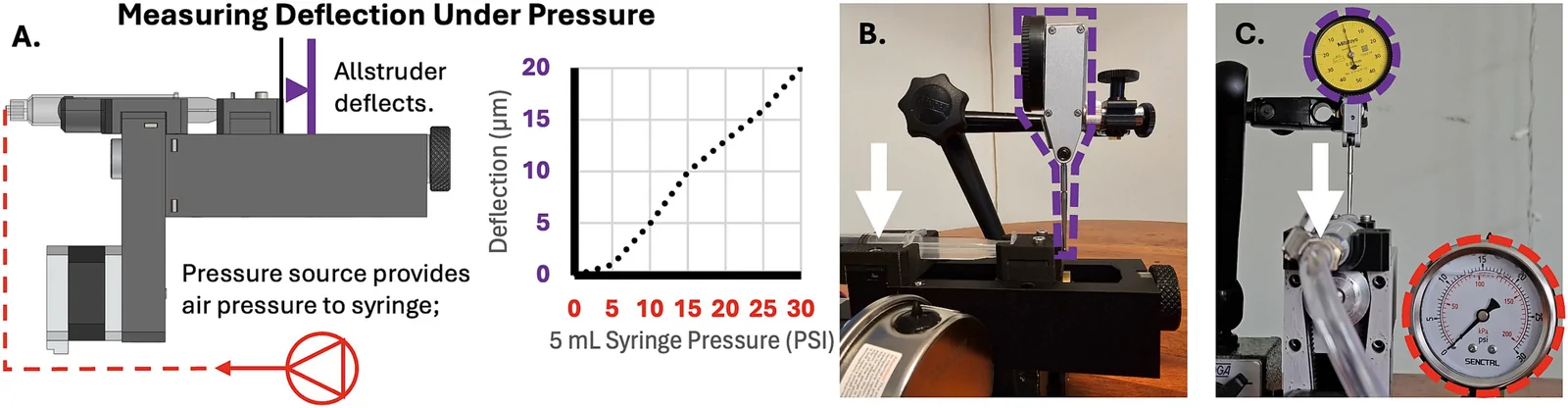
Quantifying Allstruder Deflection Under Simulated Extrusion Loading A) An air pressure source shown in red provides pressure to a 5 mL plastic syringe which causes the Allstruder to deflect; at a pressure of 30 PSI above ambient, the Allstruder deflects 20 µm B) The measurement setup is shown with a dial indicator (purple dashed lines) placed to measure the axial deflection of the Allstruder pusher when a syringe (white arrow) is pressurized. C) Measurement was accomplished by filming the dial indicator (purple dashed lines, yellow dial), the syringe (white arrow), and the syringe pressure gauge (red dashed lines) while pressure was gradually increased in the syringe. (For interpretation of the references to colour in this figure legend, the reader is referred to the web version of this article.)

The Allstruder’s actuator was mechanically tested to understand how rigid it is under loading. A Mitutoyo dial indicator was used to measure deflection of the pusher assembly when an inserted 5 mL plastic syringe was pressurized to 30 PSI or 207 kPa above ambient, which exceeds the pressure necessary to print most bioinks. For this characterization, the Allstruder’s 3D-printed components were fabricated (using settings found in the PrusaSlicer project available on the Allstruder GitHub repository) with PETG filament, 2–6 perimeters, 20% infill, and 5–7 top and bottom layers. The result (shown in Fig. 13) was a maximum deflection of 20 µm at a calculated force of 24 N. While this level of error is minimal and compounded by the compliance of plastic syringes, which introduce additional material flow under pressure, it reflects a conservative, worst-case scenario in commercial bioprinters using air-driven extrusion at maximum operating pressure. Error volume will depend on the syringe used in any given application, with larger plastic syringes having the greatest. For users requiring greater accuracy at high pressures, replacing the plastic syringe with a smaller or glass alternative would reduce deformation and improve volumetric precision.

**Fig. 14:**
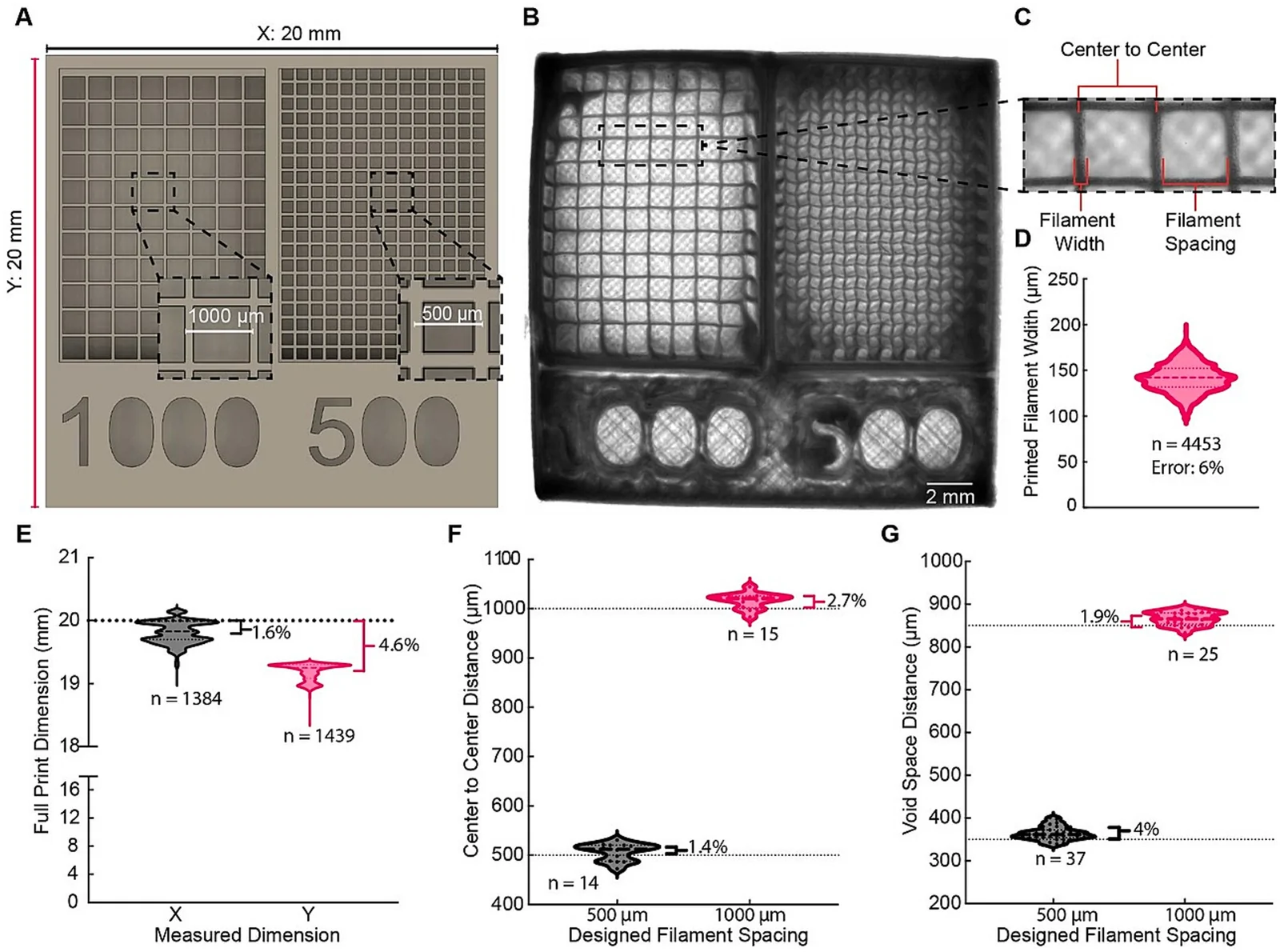
Representative Quantitative Print Performance for a Single Example Configuration. A) 3D CAD model of print resolution test. Insets highlight two different filament infill spacings tested: 1000 µm and 500 µm. B) Stereomicroscope image of printed resolution test (23 mg/mL collagen-I). C) Inset of stereomicroscope image highlighting printed single filaments with measurements taken superimposed. D) Measured width of printed collagen filament with a mean of 141.8 µm ± 15.61 µm (6% error) from the 150 µm diameter needle used. Data is presented as Mean ± SD. E) Measured width (X) and Height (Y) of entire printed construct with 19.80 ± 0.18 mm (1.6% measured print error) and 19.18 ± 0.144 mm (4.6% measured print error), respectively. Data is presented as Mean ± SD. F) Measured center to center distance of designed infill spacing 1027 ± 44 µm (2.7% measured print error from designed) for the designed 1000 µm spacing and 506.8 ± 17.30 µm (1.4% measured print error from designed) for the designed 500 µm spacing. Data is presented as Mean ± SD. G) Measured between filament spacing of 866 ± 15.84 µm (1.9% measured print error from designed) for the designed 850 µm spacing and 364 ± 15.86 µm (4% measured print error from designed) for the designed 350 µm spacing. Data is presented as Mean ± SD.

To complement the device-level deflection measurement, we quantified the output of a single complete configuration under conditions representative of ordinary use. Using the Creality K1 SE 3D printer shown in the bottom row of Fig. 12A, a 1 mL Hamilton Glass syringe, 23 mg/mL Type 1 Collagen (LifeInk® 240), and a 30 gauge x 0.5 in. needle, we printed a resolution test pattern with two distinct and common filament spacings of 1000 and 500 µm (Fig. 14A, B), and measured extruded feature width and dimensional error (Fig. 14C). Printed filament width was shown to be a mean of 141.8 μm ± 15.61 μm with a small 6% error from the 150 µm needle used for printing (Fig. 14D). The overall configuration produced results with good repeatability, and the greatest error (y axis dimension of overall print width 4.6% error) likely stems from the host printer’s mechanics and not the Allstruder (Fig. 14E). Center-to-center spacing of each spacing condition was below 3% error for each designed spacing (Fig. 14F). Spacing between each filament, which is set by subtracting the filament width (150 µm) from the designed center-to-center spacing which equates to a spacing target of 350 µm and 850 µm for 500 µm and 1000 µm, respectively, showed similar small print errors of less than 4% (Fig. 14G). As with any extrusion result, these values reflect the full configuration rather than the extruder alone. The choice of syringe is particularly influential, since barrel compliance, stiction, and seal friction vary substantially across the 0.1 to 100 mL range the Allstruder supports, and a smaller, stiffer glass syringe would be expected to yield tighter tolerances than a large plastic one. Fig. 14 therefore serves as a worked example of the Allstruder’s achievable output quality rather than a bound on it.

Together, these measurements characterize the Allstruder at two distinct levels. The deflection test in Fig. 13 quantifies the rigidity of the drive mechanism itself, a property intrinsic to the extruder that bounds achievable fidelity independently of the host printer or syringe. The resolution measurement in Fig. 14 instead quantifies the output of one complete configuration, comprising a specific host printer, syringe, material, and needle. Metrics like the latter are inherently configuration-dependent. Because the drive is a deterministic leadscrew-and-rail mechanism, the accuracy and repeatability of a finished print are governed largely by the compliance, stiction, and seal friction of the chosen syringe, by the material, and by the motion system of the host machine, rather than by the extruder in isolation. The values in Fig. 14 should therefore be read as a representative example for the stated configuration rather than as a general specification. Taken as a whole, this is the balance the Allstruder was built to strike: a mechanism rigid enough that fidelity is limited by the user's choice of syringe and printer rather than by the extruder, offered as one open and inexpensive platform that adapts to the material, the machine, and the application at hand. It is in that adaptability, demonstrated across many laboratories and quantified here at both the mechanism and the output, that the Allstruder aims to serve as shared infrastructure for the next generation of syringe-based printing.

## Ethics statements

None.

## CRediT authorship contribution statement

**Thomas Hinton:** Writing – review & editing, Writing – original draft, Visualization, Validation, Resources, Project administration, Methodology, Investigation, Formal analysis, Data curation, Conceptualization. **Riley Patten:** Writing – original draft, Visualization, Validation, Methodology, Investigation, Formal analysis, Data curation, Conceptualization. **Antonio J. PereiraTavares:** Writing – review & editing, Visualization, Investigation, Formal analysis, Data curation. **Cody Crosby:** Writing – review & editing, Validation, Investigation, Data curation. **Daniel J. Shiwarski:** Writing - review & editing, Writing - original draft, Visualization, validation, resources, project administration, methodology, investigation, formal analysis, data curation, funding support.

## Declaration of competing interest

The authors declare the following financial interests/personal relationships which may be considered as potential competing interests: T.J.H. and D.J.S. have an equity stake in FluidForm Bio, Inc, which is a startup company commercializing FRESH 3D printing. No other authors declare that they have known competing financial interests or personal relationships that could have appeared to influence the work reported in this paper.

## Appendix A Supplementary data

Data within the supplementary files include: CAD files, assembly instructions, slicing profiles, printer mounting hardware, and additional resources. All other information can be found at the Allstruder Github page and Zenodo deposit. The following are the Supplementary data to this article:
